# Ligand-Induced Structural Evolution and Luminescence Tuning in a Series of Superatomic Ir/Ag Hydride-Containing Nanoclusters

**DOI:** 10.1021/acs.inorgchem.6c01668

**Published:** 2026-06-19

**Authors:** Wei-Jung Yen, Tzu-Hao Chiu, Michael N. Pillay, Samia Kahlal, Jean-Yves Saillard, C. W. Liu

**Affiliations:** † Department of Chemistry, 63373 National Dong Hwa University, Hualien 97401, Taiwan Republic of China; ‡ Univ Rennes, CNRS, ISCR-UMR 6226, F-35000 Rennes, France

## Abstract

A series of hydride-containing bimetallic 8-electron silver-rich superatomic nanoclusters doped with IrH, formulated as IrHAg_20_[E_2_PR_2_]_12_ (E = S, Se; R = O^*n*^Pr, O^*i*^Pr, Ph), were synthesized via a one-pot strategy in combination with ligand-exchange-induced structure transformation (LEIST), which enabled the generation of capping-atom isomers. Single-crystal X-ray diffraction revealed that modification of the ligand shell drives capping-atom migration, leading to structural diversity with symmetries ranging from *C*_1_ to *C*_3_. Among them, IrHAg_20_[Se_2_P­(O^*i*^Pr)_2_]_12_ represents the first Ir/Ag superatomic nanocluster stabilized by Se-donor ligands, thereby expanding the examples of ligand-protected silver-rich superatoms. All four compounds exhibit solid-state PL in the NIR region, with quantum yields reaching up to 10%. Notably, IrHAg_20_[S_2_P­(O^*i*^Pr)_2_]_12_ displays the highest PLQY. This enhanced emission is attributed to the asymmetric passivating ligand shell, which induces greater distortion of the superatomic core, thereby increasing the transition dipole moment and leading to a pronounced enhancement of PL intensity. Collectively, these findings highlight the critical role of ligand-induced strain and intramolecular interactions in modulating the luminescence of silver-rich nanoclusters, offering new design principles for emissive superatomic materials.

## Graphical abstract

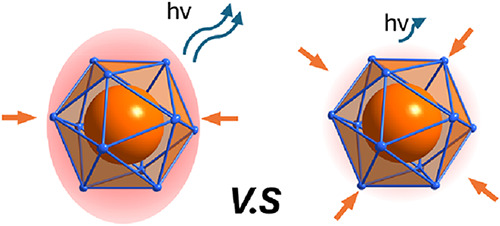

## Introduction

Ligand-protected coinage metal nanoclusters (NCs) have emerged as a fascinating class of materials due to their atomically precise structures and unique electronic features that bridge the gap between discrete metal complexes and bulk nanomaterials. Over the past two decades, these NCs have been extensively studied not only for their fundamental scientific value but also for their broad application potential in catalysis, sensing, bioimaging, and optoelectronics.^1–7^ Their well-defined structures allow the establishment of clear correlations between atomic arrangement and properties, providing an ideal platform for mechanistic understanding and rational material design. Moreover, tuning and enhancing the NIR emission properties of the NCs represent critical research directions, aiming not only to improve optical performance but also to achieve multifunctional capabilities.^8–11^

A key focus in NCs research has been the rational tuning of their optical properties. Two general strategies have been widely adopted: (i) heterometal doping, which introduces electronic perturbations into the metallic framework, and (ii) ligand modification, which alters the surface of the metallic core and the cluster rigidity. For example, Tsukuda and co-workers demonstrated that doping an Au_13_ NC with Ir resulted in highly emissive IrAu_12_ clusters,^12–14^ and the use of rigid ligands further boosted their photoluminescence quantum yield (PLQY) to an impressive value of 87%.^15^ In silver-rich systems, Lee and his colleagues showed that incorporation of group 8–9 transition metals into Ag_25_ superatoms generated intriguing PLQY trends, largely modulated by the presence of interstitial hydrides.^16,17^ Beyond doping, ligand-exchange-induced structure transformation (LEIST)^18^ has become a powerful strategy for manipulating surface chemistry, structural isomerization, and emission intensity, as exemplified by PtAg_28_(S-c-C_6_H_11_)_18_(PPh_3_)_4_,^19^ which underwent structural isomerization and enhanced fluorescence upon ligand exchange with adamantanethiolate. Although these advances highlight the importance of heterometal doping and ligand-exchange strategies, the influence of isomerization-induced distortion of the superatomic core on photoluminescence intensity has been rarely explored. Most recent studies have focused on changes in electronic configuration or rigidity imparted by ligand frameworks, while overlooking the possibility that high structural symmetry may suppress the transition dipole moment and consequently reduce emission intensity. Most recently, our group isolated and characterized IrH_2_Ag_19_[S_2_P­(O^*n*^Pr)_2_]_12_ and (IrH_2_)_4_Ag_50_[S_2_P­(O^*n*^Pr)_2_]_22_,^20^ demonstrating the ability of the Ir-doped systems to form superclusters.

In this work, we present four well-defined hydride-containing Ir/Ag 8-electron superatomic NCs stabilized by dichalcogenide ligands: IrHAg_20_L_12_ (L = [S_2_P­(O^*i*^Pr)_2_]^−^ (**1a**), [S_2_P­(O^*n*^Pr)_2_]^−^ (**1b**), (S_2_PPh_2_)^−^ (**1c**), and [Se_2_P­(O^*i*^Pr)_2_]^−^ (**2**)). Single-crystal X-ray diffraction reveals diverse structural motifs with symmetries ranging from *C*_1_ to *C*_3_. Among them, **1a**, which exhibits the lowest symmetry, is particularly notable, as the distortion of its superatomic core results in a larger transition dipole moment, correlating with a markedly enhanced PLQY. In addition, compound **2** represents the first 8e Ir/Ag superatomic NCs stabilized by Se-donor ligands, diselenophosphate (dsep), with compounds **1a**–**c** reported to be dithiolate-stabilized 8e Ir/Ag superatoms, thereby expanding the ligand chemistry available for silver-rich superatoms. Overall, these results underscore the importance of superatomic core distortion in governing emission behavior and provide new examples of luminescent noble metal NCs.

## Results and Discussion

### Synthesis and Spectroscopy Analysis

The synthesis follows our previously established pathway for MAg_20_[dtp]_12_ (M = Pd,^21,22^ Pt;^23,24^ dtp = dithiophosphate) and MHAg_19_[dtp]_12_ (M = Pd,^25,26^ Pt^27^) superatomic NCs. As outlined in Scheme 1, compounds **1a** and **1b** were obtained via a coreduction method, and the orange fraction collected after column chromatography purification yielded the pure products in 17.37% yield. Compounds **1c** and **2** were subsequently generated by the LEIST method by treating the precursors with 12 equiv of K­[S_2_PPh_2_] or [NH_4_]­[Se_2_P­(O^*i*^Pr)_2_] in a THF solution at 0 °C. During the course of the reaction, the solution color changed from dark brown to bright orange. After rotary evaporation, the crude products were further purified by extraction to remove residual dtp ligands, affording the final products in isolated yields of 81.2% (**1c**) and 79.8% (**2**) following vacuum drying. Electrospray ionization mass spectroscopy (ESI-MS) was applied to determine the composition of **1a**, **1b**, **1c**, and **2**. The positive-mode spectrum shows that both compounds **1a** and **1b** show a peak at *m*/*z* = 5017.4326 Da, consistent with the theoretical molecular ion peak [**1a**+Ag]^+^ or [**1b**+Ag]^+^ (Sim.: 5017.1770 Da, Figure 1a). After ligand exchange, the positive-mode spectrum reveals a peak at 5450.9917 Da for **1c** and 6143.8604 Da for **2**, which fit to the theoretical molecular ion peaks [**1c**+Ag]^+^ (Sim.: 5449.9268 Da) and [**2**+Ag]^+^ (Sim.: 6143.8701 Da) by the ESI-mass (Figure 1b and Figure S1). The deuteride analogue **1a**_**D**_ was detected with a 1 Da mass-unit difference (Figure S2), further confirming the assigned composition of **1a**.

**Figure 1:**
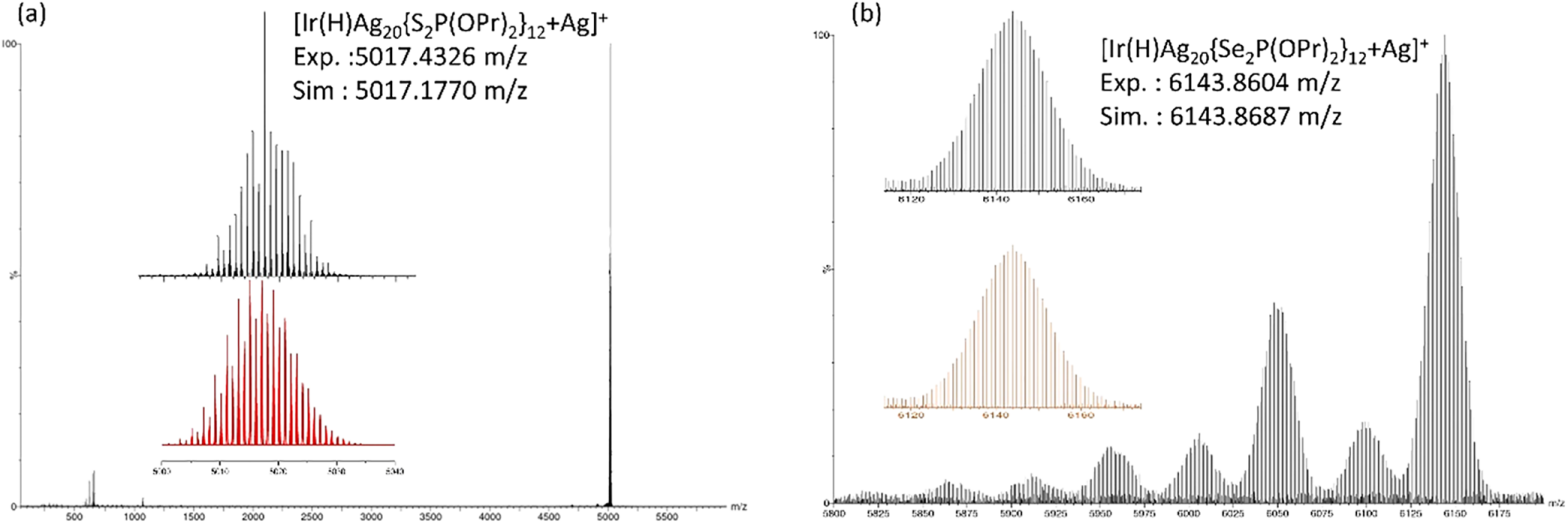
(a) ESI-MS spectrum of **1a** showing the molecular ion peak corresponding to [**1a**+Ag]^+^. (b) The ESI-MS spectrum of **2** showing the molecular ion peak corresponding to [**2**+Ag]^+^.

**Scheme 1:**
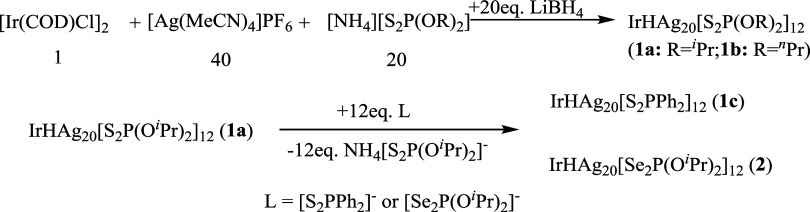
Synthesis of IrHAg_20_L_12_

The solution dynamics of the NCs were evaluated by NMR spectroscopy. Integration of the ligand shell protons relative to the hydride signals confirms the assigned 12:1 ligand-to-hydride ratio in all cases. Across the series, the observed resonance for the encapsulated hydride spans a narrow range from −16.85 to −17.66 ppm (Figures S7–S10), marginally upfield in comparison to the thiolate-protected [IrHAg_24_(SR)_18_]^2–^ (−14 ppm).^16^ At ambient conditions, the ^31^P NMR spectra of **1a**, **1b**, and **1c** display sharp resonances at 101, 104, and 65 ppm, respectively (Figures S3–S5), while **2** exhibits a relatively broad resonance centered at 67 ppm (Figure S6). Variable-temperature NMR experiments further elucidated the fluxional behavior of the ligand and hydride environments. At 193 K, the spectra of **1a** appear significantly more complex than the more symmetric counterparts **1b** and **1c**. The ^31^P NMR spectrum of **1a** shows four prominent resonances along with several minor peaks, indicating the presence of multiple isomers in solution. This fluxionality is reminiscent of the behavior previously observed in related silver-rich systems [PdHAg_19_(dtp)_12_] and [RhH_*x*_Ag_21–*x*_{S_2_P­(O^n^Pr)_2_}_12_] (*x* = 0–2)^25,28^ (Figure S11). Furthermore, the low-temperature ^1^H NMR spectrum indicates three distinct hydride environments and resonances corresponding to different isomers. The persistence of both the ligand shell and the hydride fluxionality even at low temperatures is apparent; however, one of the isomers exhibits a characteristic quartet pattern arising from scalar coupling with ^107^Ag/^109^Ag nuclei (Figure S12). In contrast, for **1b**, upon cooling to 213 K, the ^31^P NMR resonance splits into four signals of equal intensity, corresponding to the four distinct ligand environments expected for a *C*_3_-symmetric structure (Figure S13). The ^1^H NMR spectrum of **1b** at 193 K is consistent with the hydride coupling with three silver nuclei, comparable to those observed for [PtHAg_19_(dtp)_12_][Bibr ref27] and [PdHAg_19_(dtp)_12_][Bibr ref25] (Figure S14). Further confirmation of the assigned hydride position was obtained via the deuteride analogues **1a**_**D**_ and **1b**_**D**_, which were prepared by substituting NaBH_4_ with NaBD_4_. The ^31^P NMR chemical shifts for **1a**_**D**_ and **1b**_**D**_ remained consistent, with sharp resonances observed at 101 and 104 ppm, respectively (Figures S15 and S16). Crucially, the ^2^H NMR spectra display single peaks at −17.24 and −17.47 ppm (Figures S17 and S18), for **1a**_**D**_ and **1b**_**D**_, respectively, which are in full agreement with the ^1^H NMR spectra of **1a** and **1b**.

### Single-Crystal X-ray Diffraction Analysis

Compounds **1a**, **1b**, and **2** were recrystallized from MeOH solution, whereas **1c** was recrystallized from dichloromethane due to its limited solubility. The X-ray structures of **1** and **2** are depicted in Figure 2, and selected bond distances and angles are given in Table 1. They reveal that both **1** and **2** possess a distorted centered icosahedral [(IrH)@(Ag_ico_)_12_] kernel, passivated by 8 capping Ag atoms (Ag_cap_) and 12 dtp or dsep ligands. Each Ag_cap_ is located above a triangular face of the central Ag_12_ icosahedron and further coordinated by three S or Se from the ligands. This structural feature is that of 8-electron [(IrH)@Ag_12_]^4+^ superatomic cores protected by a [Ag_8_(dtp/dsep)_12_]^4–^ passivating layer in which the Ag_cap_ atoms are in their +I oxidation state.^24–31^ In the case of **1a**, the IrHAg_20_ framework adopts a *C*_1_ symmetry, where the 8 Ag_cap_ atoms are divided into three groups with a 3:3:2 distribution (Figure 2b). This arrangement is reminiscent of the reported PdAg_20_ and PtAg_20_ clusters.^21–24^ By contrast, **1b** and **2** share a similar metal framework of idealized *C*_3_ symmetry, which derives from the *D*_3_ arrangement of Ag_21_ ^29^ by a lowering of symmetry caused by the presence of the interstitial hydride (Figure 2c). In **1c**, the 8 Ag_cap_ are regularly located on eight nonadjacent triangular faces of the icosahedron, forming a giant cube, as in the previously reported MAg_20_(dsep)_12_ clusters (M = Ag, Au, Pd, Pt), whose metal framework idealized symmetry is *T* ^21,25,30,31^ (Figure 2d). However, the presence of an interstitial hydride in **1c** lowers its IrHAg_20_ idealized symmetry to *C*_3_. This *C*_3_ structure is similar to one of the previously predicted *C*_3_ isomers of [PdHAg_20_(dtp)_12_]^+^.^25^ Because the eight capping Ag atoms in **1b**, **1c**, and **2** are more evenly distributed over the triangular faces, the resulting IrHAg_12_ kernels appear more spherical, with narrower Ir–Ag distance distributions. In contrast, the IrHAg_12_ kernel of **1a** is slightly elongated into an ellipsoid, with its Ag_cap_ preferentially arranged along the short axis (Figure 3). This distinction is reflected in the Ir–Ag bond distances: in **1a**, the broader distance distribution indicates a compressed icosahedron into an ellipsoid by the surrounding passivating layer. Analysis of the continuous shape measure (CSM)^32^ further supports this observation, with **1a** showing the largest degree of distortion and **1b** the least, although all structures exhibit some extent of distortion, similar to previously reported 8-electron superatoms containing interstitial hydrides. In fact, both **1a** and **1b** are structural isomers,^6^ rarely observed in the superatomic alloys. A notable difference arises in **1c**, where the ligands, unlike the fully saturated alkyl groups in the other three clusters, contain aromatic rings. Similar to previously reported examples bearing aromatic ligands, several weak interactions can be observed between the ligands.^33–35^ Such noncovalent interactions not only enhance structural stability but also suppress nonradiative decay pathways in photophysical processes. Within **1c**, intramolecular C–H···π interactions are clearly identified, leading to a nonequilateral triangular configuration where three phenyl rings are held together through C–H···π interactions (2.96–3.4 Å) (Figure S18).

**Figure 2:**
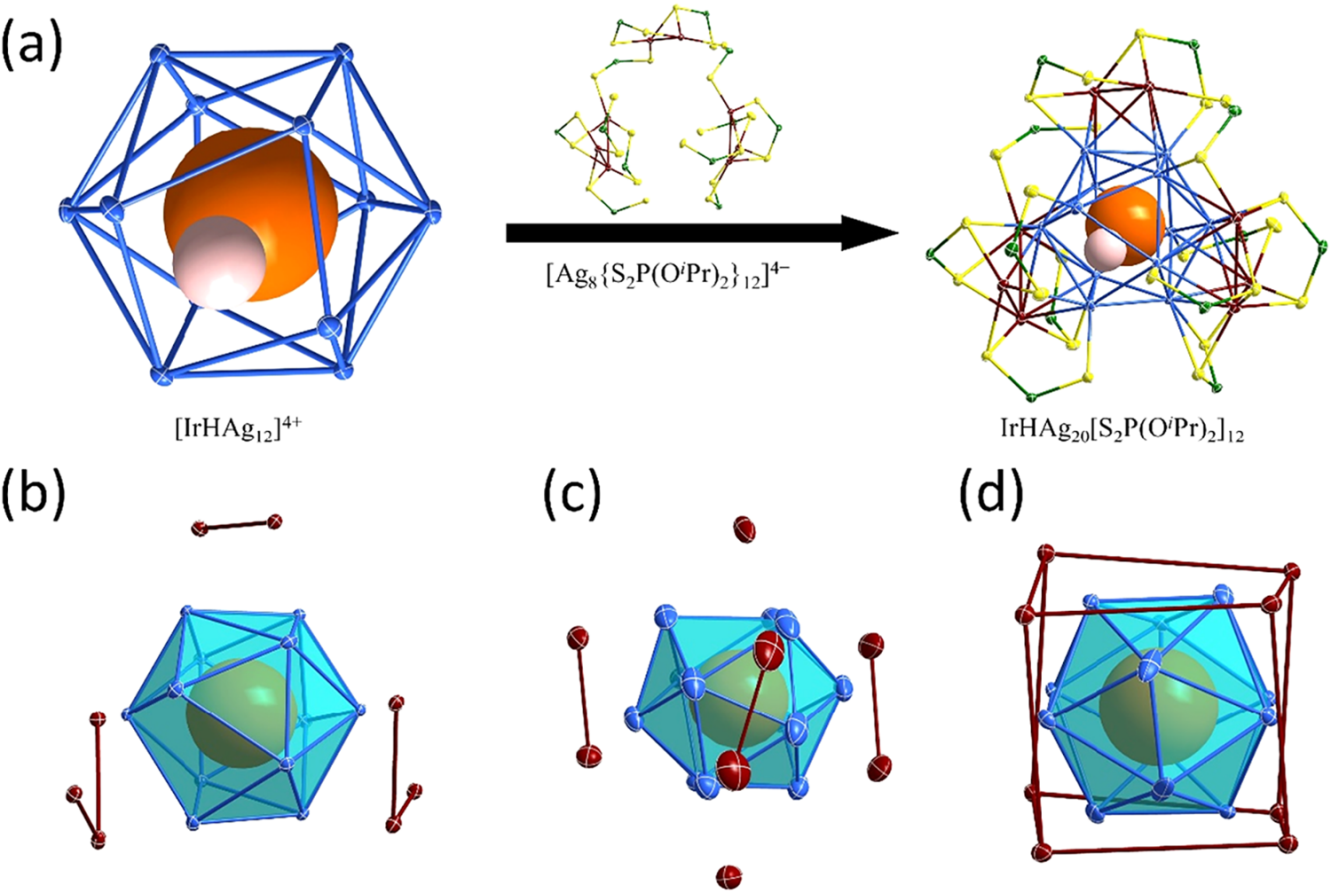
(a) Structure of **1a**, the isopropyl groups are omitted for clarity. The metal framework of (b) **1b**, (c) **1c**, and (d) **2** (color code: Ag_ico_, blue; Ag_cap_, brown; S, yellow; P, green; Ir/IrH, orange; H, pink).

**Figure 3:**
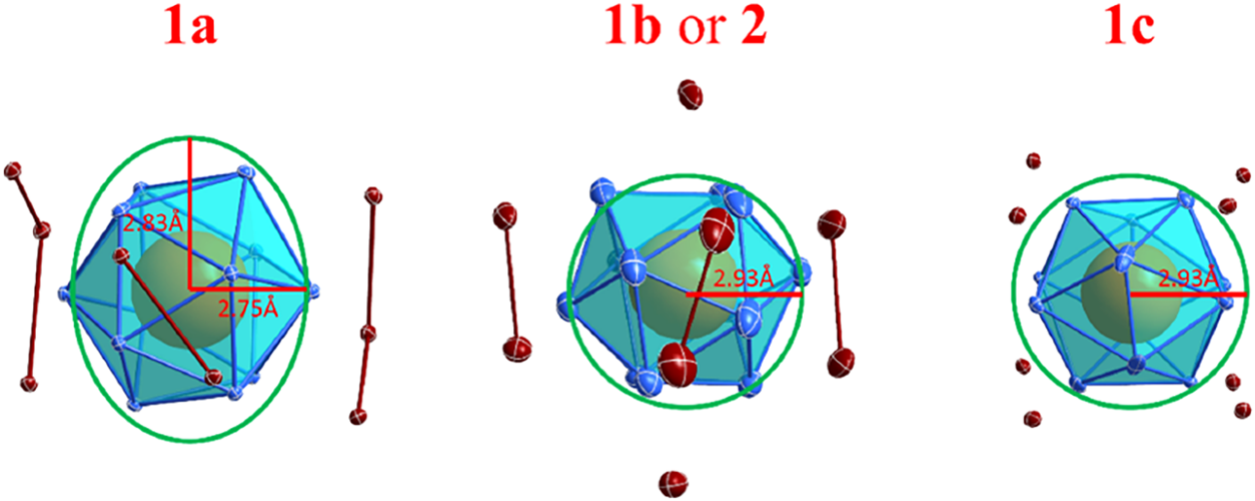
Metal framework of **1**–**2**.

**Table 1:** Selected Experimental Bond Distances of **1a**, **1b**, **1c**, and **2**

	**1a**	**1b**	**1c**	**2**
CSM	0.336	0.166	0.194	0.306
Ir–Ag_ico_	2.706(2)–2.954(3) avg. 2.792(2)	2.748(2)–2.833(2) avg. 2.786(2)	2.750(2)–2.868(3) avg. 2.787(2)	2.740(2)–2.856(1) avg. 2.785(2)
Ag_ico_–Ag_ico_	2.776(2)–3.658(4) avg. 2.936(2)	2.791(2)–3.242(3) avg. 2.930(2)	2.775(2)–3.431(2) avg. 2.928(2)	2.773(1)–3.476(3) avg. 2.929(2)
Ag_ico_–Ag_cap_	2.852(2)–3.208(2) avg. 2.989(2)	2.747(2)–3.150(2) avg. 2.974(2)	2.910(3)–3.123(2) avg. 2.979(2)	2.848(2)–3.133(2) avg. 2.981(2)

A comparison of the icosahedra in [Ir@Au_12_] ^12–15^ and the current Ag-series reveals a more compact kernel in the former, leading to inherent stabilization of the central Ir atom without the need for additional support. In the current report, the less electronegative silver framework instead utilizes an interstitial hydride (H^–^) to provide localized electron density to meet the electronic requirements of the central iridium core. The preference of the silver core to encapsulate a hydride rather than altering the overall cluster charge state can be rationalized by formal cluster electron counting and charge-neutrality principles. If the silver system were to avoid hydride encapsulation and instead emulate the gold system, maintenance of charge neutrality would require an additional silver atom in the passivation layer. This would effectively force a structural shift from the IrH@Ag_20_ core to a hypothetical Ir@Ag_21_. This nuclearity-dependent framework compensation to preserve a closed electronic shell correlates with the trend previously observed in the structurally related Rh/Ag system, where varying the hydride count directly correlates with the addition or removal of capping silver atoms in the [RhH_*x*_@Ag_21–*x*_(dtp)_12_] (*x* = 0–2) series.^28^

### X-ray Photoelectron Spectroscopy Analysis

The X-ray photoelectron spectroscopy (XPS) studies were performed to evaluate the metal oxidation state in **1a**–**2** by analyzing their respective binding energies (BEs) (Figure S19). The Ag (3d_5/2_) BEs of **1a**–**2** fall between those of Ag(0) and Ag­(I),^36^ suggesting that the Ag atoms exhibit an averaged mixed-valence character (Figure S20). In addition, the Ir 4f_7/2_ BEs of **1a**–**1c** are shifted to a lower binding energy relative to the metallic Ir foil (Figure S21), indicating that the Ir centers are more electron-rich than Ir(0),^36^ in full agreement with the DFT-computed Ir atomic charges (see below).^37^

### Photophysical Properties

All four NCs exhibit absorption features around 400 nm. **1a** and **1b** display nearly identical absorption maxima (**1a**: 396 nm; **1b**: 395 nm). In contrast, **1c** and **2** show two distinct absorption bands at 382 and 436 nm for **1c** and 385 and 433 nm for **2** (Figure S22). Diffuse reflectance spectroscopy primarily provides information on the optical band gap of compounds **1a**–**2** to elucidate the optical band gap in the solid state, which is consistent with DPV spectra (*vide infra*, Figures S23 and S24). The four NCs exhibit NIR photoluminescence (PL) in both the solid and the film states (Figure 4). Their solid-state spectra exhibit pronounced differences that can be rationalized by considering both radiative and nonradiative decay pathways (Table 2). **1a** exhibits the highest PLQY (7.53%), which is primarily attributed to its highly distorted superatomic core arising from its lowest symmetry. In contrast, the most symmetrical **1b**, **1c**, and **2** display lower PLQYs overall, indicating that reduced core distortion limits the enhancement of radiative transitions. Within this series of structurally related clusters, however, noticeable differences in PLQY are still observed, suggesting that secondary factors play an important role in modulating their emission efficiencies. Notably, among the *C*_3_-symmetric clusters, compound **1c** exhibits a relatively enhanced PLQY and the longest lifetime. This improvement is attributed to the presence of diphenyl ligands, which introduce intramolecular C–H···π interactions that effectively rigidify the ligand shell and reduce nonradiative decay pathways (Figure S25). By comparison, the more flexible alkyl-substituted ligands in **1b** and the Se-donor system in **2** provide fewer opportunities for such intramolecular locking, resulting in larger nonradiative decay rate constants and diminished PLQY. These observations demonstrate that while superatomic core distortion governs the overall emission strength, ligand-induced intramolecular interactions serve as an effective secondary handle to fine-tune PL within clusters of comparable symmetry.

**Figure 4:**
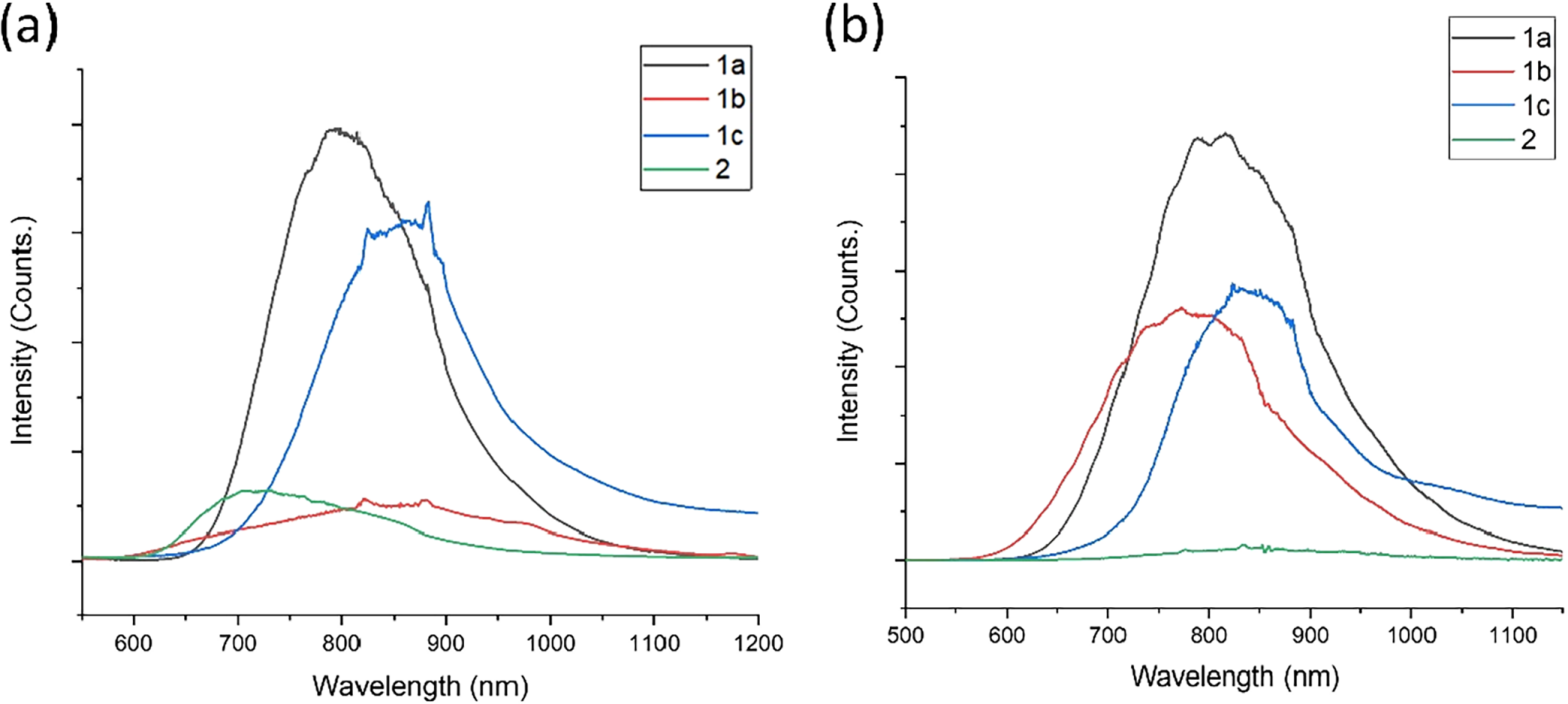
PL spectra of **1a**, **1b**, **1c**, and **2**: (a) solid state; (b) film state.

**Table 2:** Photophysical Properties of **1a**, **1b**, **1c**, and **2**

	**1a**	**1b**	**1c**	**2**
Solid				
Emission	790	805	860	720
Lifetime (μs, avg.)	533.91	109.64	631.18	140.28
PLQY	7.53%	0.51%	1.93%	0.40%
*k*_*r*_	1.41 × 10^2^ s^–1^	4.65 × 10^1^ s^–1^	3.06 × 10^1^ s^–1^	2.85 × 10^1^ s^–1^
*k*_*nr*_	1.73 × 10^3^ s^–1^	9.11 × 10^3^ s^–1^	1.56 × 10^3^ s^–1^	7.11 × 10^3^ s^–1^
Film				
Emission	790	772	826	845
Lifetime (μs, avg.)	8.44	9.25	11.37	12.57
PLQY	10.29%	4.24%	5.35%	1.92%
*k*_*r*_	1.22 × 10^4^ s^–1^	4.58 × 10^3^ s^–1^	4.71 × 10^3^ s^–1^	1.53 × 10^3^ s^–1^
*k*_*nr*_	1.06 × 10^5^ s^–1^	1.04 × 10^5^ s^–1^	8.28 × 10^4^ s^–1^	7.83 × 10^4^ s^–1^

Upon fabrication into thin films, all four clusters exhibit significantly enhanced PLQY accompanied by a decrease of the excited-state lifetimes, reflecting an influence of the cluster environment. Importantly, the relative PLQY ordering among the *C*_3_-type clusters is largely preserved in the film state, with **1c** remaining more emissive than **1b** and **2**. This trend further confirms that the enhanced emission of **1c** originates from intrinsic ligand-induced rigidity rather than crystal packing effects. Together with the dominant role of core distortion observed in **1a**, these results establish a hierarchical structure–property relationship in which superatomic core symmetry dictates the primary emission efficiency, while ligand engineering provides a secondary means to optimize luminescence.

In comparison to the studies on the photoluminescent properties in Ir/Au systems,^12–15^ Ir/Ag systems^16^ remain notably fewer, particularly in the solid state. In addition to the geometric and electronic differences, there is a distinct mechanistic difference in the PLQY-enhancement mechanism. While the gold-rich system primarily relies on kernel rigidification to suppress nonradiative energy loss,^14^ the current silver-rich series utilizes core distortion to increase the radiative transition probability. Thus, the primary driver for PLQY enhancement is an acceleration of the radiative rate (*k*_r_). In contrast to the *C*_3_ clusters **1b** and **1c**, where the transition dipole moment is canceled by the selection rules, the different core distortion in **1a** activates the transition dipole moment, thereby enhancing its PLQY relative to **1b** and **1c**.

### Electrochemical Properties

Differential pulse voltammetry (DPV) measurements were performed in dichloromethane at 253 K (Figure S26, Table S1). All clusters exhibit multiple oxidation and reduction events, reflecting stepwise redox processes of the superatom. The first reduction waves appear between −2.24 and −1.82 V, with the diphenyl-substituted cluster showing a notably less negative reduction potential, indicative of enhanced electron-accepting ability induced by aromatic ligands. In contrast, the electrochemical gaps derived from the onset potentials remain comparable across the series, suggesting that ligand exchange does not alter the underlying 8-electron superatomic configuration but instead subtly modulates the frontier orbital energies. These results indicate that the observed differences in photophysical properties arise primarily from structural distortion and ligand-induced suppression of nonradiative decay rather than changes in the intrinsic electronic gap.

### DFT Computational Analysis

In order to get a better insight into the electronic structures of the four NCs, DFT calculations were performed at the BP86/Def2-TZVP level (computational details in the Experimental Section below) on four models in which the dichalcogenide ligands have been replaced by the simpler S_2_PH_2_ or Se_2_PH_2_, a modeling that has been fully validated in many past investigations,^22,24–31^ while allowing considerable CPU time saving. Note that this simplification leads to the same composition for models **1a**, **1b**, and **1c**, making them true isomers. Relevant DFT-computed data are provided in Table 3. The optimized geometries are in good agreement with their experimental counterparts, in particular, with respect to the icosahedral distortions and hydride locations. A reasonable displacement of the hydride from its equilibrium position, followed by a full geometry reoptimization, results in its return to its equilibrium position, thus bringing even more confidence to our results. The small computed values of the Ag_ico_–Ag_cap_ Wiberg bond indices (WBIs) are consistent with metallophilic interactions, whereas the Ir–Ag_ico_ ones indicate non-negligible covalency. Within their IrAg_3_ tetrahedra, the hydrides are strongly bonded to Ir and weakly to Ag, as shown by the corresponding WBI values. The non-negligible Ir–H covalent bonding is reflected by the not-so-negative value of the hydride atomic charges. All of these results are consistent with those obtained previously for the isoelectronic [RhHAg_20_(dtp)_12_] relative.^28^ Our **1a**, **1b**, and **1c** models being isomers, one can compare the relative stability of their different architectures. **1b** is found to be the most stable, both from the point of view of the total (*E*) and free (*G*) energies (Table 3). However, the two other isomers are found less stable by only a few kcal·mol^–1^. These results are consistent with previous investigations on related 8-electron isoelectronic systems with the superatomic 1S^2^ 1P^6^ 1D^0^ electronic configuration of the [(IrH)@Ag_12_]^4+^ core in which the IrH unit contributes 0 electron and Ag(0) 1 electron.^25,28,35^ This 8-electron core is further capped by 8 Ag^+^ metal centers and 12 dtp^–^ ligands. Consistently, the three highest occupied orbitals of the four NCs can be identified as the 1P ones, whereas the 1D manifold can be associated with the five lowest, or lying among the six lowest, orbitals. A selection of these frontier orbitals is plotted in Figures S27–S30, and the Kohn–Sham orbital diagrams of **1a**, **1b**, and **1c** are provided in Figure S31.

**Table 3:** Relevant Data Computed for the Models of **1a**, **1b**, **1c**, and **2** ^a^

		**1a**	**1b**	**1c**	**2**
Δ*E*_HOMO–LUMO_ (eV)		1.74	1.94	2.17	1.72
Δ*E* (kcal·mol^–1^)		2.9	0.0	2.9	-
Δ*G* (kcal·mol^–1^)		1.3	0.0	5.1	-
CSM		0.320	0.275	0.258	0.280
Ir–Ag_ico_ (avg.)		2.880 [0.165]	2.881 [0.164]	2.878 [0.165]	2.884 [0.155]
Ag_ico_–Ag_ico_ (avg.)		3.029 [0.075]	3.027 [0.077]	2.977 [0.077]	3.031 [0.076]
Ag_ico_–Ag_cap_ (avg.)		3.112 [0.047]	3.072 [0.044]	3.065 [0.039]	3.126 [0.043]
Ir–H		1.673 [0.265]	1.649 [0.304]	1.676 [0.238]	1.651 [0.300]
Ag–H (avg.)		2.060 [0.097]	2.073 [0.087]	2.044 [0.110]	2.080 [0.086]
NPA atomic charges	Ir	–1.66	–1.66	–1.66	–1.62
	H	–0.34	–0.31	–0.36	–0.31
	Ag_ico_	0.35	0.35	0.34	0.33
	Ag_cap_	0.69	0.70	0.71	0.65
λ_max_ (nm)		377	337	341	354
			409	392	438

a Bond distances are given in Å. The associated Wiberg bond indices (WBIs) are provided in brackets. NPA = natural population analysis.

Single-point TD-DFT calculations were performed at the CAM-B3LYP/Def2-TZVP level (see the computational details in SI) to simulate the UV–vis absorption spectra of the four NCs, which are shown in Figure S32, with their λ_max_ reported in Table 3. They are in good agreement with their experimental homologues. The bands of lower energy are of 1P → 1D character (Table S3 and Figure S31), whereas the band at higher energy is of mixed 1P → 1D and 1P → ligand nature. Looking at the largest wavelengths in the case of **1a**, it is interesting to note that the first non-negligible oscillator strength (at 483 nm, see Table S3) is particularly large and contributes significantly to the band tail. Although doubly degenerate, this first electronic transition in **1b**, **1c**, and **2** (at 459, 421, and 490 nm, respectively) is associated with a substantially lower oscillator strength, in particular, in the case of **1b** and **1c**. It is likely that the corresponding singlet excited state is the one involved in the PL process, in agreement with the fact that **1a** has, by far, the largest PLQY. On the other hand, the particularly low PLQY of **2** should also be the consequence of a more important nonradiative decay due to the floppier dsep ligand. In the four NCs, this first low energy transitions are of HOMO and HOMO–1 → LUMO nature, but in the case of **1a**, it is mixed with some (16%) HOMO–4 → LUMO character, the HOMO–4 being of large 5d­(Ir) character. This mixing is allowed by the specific topology of the *C*_1_ architecture of **1a**, conferring to the latter a larger transition dipole moment.

## Concluding Remarks

In summary, we have synthesized four new hydride-containing 8-electron Ir/Ag superatoms. In addition to employing a one-pot synthetic strategy, ligand substitution enables the formation of pseudoisomers, ultimately affording the first Ir/Ag superatom stabilized by a selenium-donor ligand, thereby expanding the scope of ligand protection chemistry in silver-rich clusters. Moreover, all solid-state samples exhibit NIR PL. Among them, compound **1a**, which features the most distorted superatomic core, displays the highest PLQY. Furthermore, among the three clusters of *C*_3_ symmetry, weak intramolecular interactions within the passivating ligand shell effectively reduce energy dissipation, thus enhancing emission. Collectively, these results highlight the critical roles of superatomic core distortion and intramolecular interactions in governing cluster luminescence and provide new insights into the rational design of emissive coinage metal NCs through ligand engineering.

## Experimental Section

### General Remarks

All chemicals were purchased from commercial sources and used as received. Solvents were purified following standard protocols. All reactions were carried out under N_2_ atmosphere by using standard Schlenk techniques. [Ag­(CH_3_CN)_4_]­PF_6_ and NH_4_[S_2_P­(O^*n*^Pr)_2_] were prepared by following the procedure reported in the literature.^38,39^ NMR spectra were recorded on a Bruker AvanceNeo 500 MHz NMR spectrometer and a JEOL ECZL-R series 500 MHz NMR spectrometer. The chemical shift (δ) is reported in ppm, with ^1^H spectra referenced to residual solvent, and ^31^P chemical shift (δ) referenced to an H_3_PO_4_ external standard. ESI-mass spectra were recorded on a Bruker maXis Q-TOF mass spectrometer (Bruker Daltonik GmbH, Germany). UV–visible–NIR absorption spectra were measured on a Cary60 spectrophotometer at 298 K, using quartz cells with a path length of 1 cm. The solid-state diffuse reflection spectra were collected on a Shimadzu UV-2600i with an integrating sphere. Luminescence spectra and lifetime were recorded on a HORIBA JOBIN YVON FluoroMax^+^ spectrometer with PMT-R13456 and InGaAs-1700 (for the NIR) detector head mounted on the exit port. X-ray photoelectron spectroscopy (XPS) spectra were recorded on a spectrometer (VG Multilab 2000-Thermo Scientific Inc., UK, Kα) with a microfocus monochromated Al Kα X-ray working with high photonic energies from 0.2 to 3 keV.

### Synthesis

#### Synthesis of [IrHAg_20_{S_2_P­(O^*i*^Pr)_2_}_12_] (**1a**)

In a Schlenk tube, [Ag­(CH_3_CN)_4_]­PF_6_ (0.50 g, 0.86 mmol) and NH_4_[S_2_P­(O^*i*^Pr)_2_] (0.14 g, 0.43 mmol) were dissolved in THF and stirred at −20 °C for 5 min. [Ir­(COD)­Cl]_2_ (0.02 g, 0.043 mmol) was then added and after 10 min, LiBH_4_ (0.6 mL, 2 M THF solution, 1.2 mmol) was added and stirred for 1 day. The reaction mixture was dried under reduced pressure. The residue was extracted with DCM and washed with DI water. The DCM layer was dried under reduced pressure and then purified by Al_2_O_3_ column chromatography to yield **1a** (25.4 mg, 17.37% based on Ag). ^31^P­{^1^H} NMR (202.468 MHz, CDCl_3_, δ, ppm, r.t.): 101.4 ^1^H NMR (500.15 MHz, THF-*d*_8_, δ, ppm, r.t.): −17.3 (br, μ_4_-H, 1H), 1.40 (t, CH_3_, 144H), 4.99 (m, OCH, 24H). ^1^H NMR (500.15 MHz, THF-*d*_8_, δ, ppm, 193 K): −17.72 (q, μ_4_-H, 1H, ^1^ *J*_1H‑107Ag_ = 29.7 Hz, ^1^ *J*_1H‑109Ag_ = 34.2 Hz), −16.82 (br, μ_4_-H, 1H), −16.24 (br, μ_4_-H, 1H). ESI-MS (*m*/*z*): exp. 5017.4326 (calc. for [M + Ag^+^]^+^: 5017.1770). UV–vis [λ_max_ in nm (ε in M^–1^ cm^–1^)]: 396 (57800).

#### Synthesis of [IrHAg_20_{S_2_P­(O^*n*^Pr)_2_}_12_] (**1b**)

An analogous procedure to **1a** except for the substitution of the ligand for NH_4_[S_2_P­(O^*n*^Pr)_2_]. **1b** (Yield: 35.2 mg, 24.08% based on Ag). ^31^P­{^1^H} NMR (202.468 MHz, CDCl_3_, δ, ppm, r.t.): 104.2 ^1^H NMR (500.15 MHz, THF-*d*_8_, δ, ppm, r.t.): −17.6 (br, μ_4_-H, 1H), 0.97 (t, CH_3_, 72H), 1.74 (septet, CH_2_, 48H), 4.08 (q, OCH_2_, 48H). ^31^P­{^1^H} NMR (202.468 MHz, THF-*d*_8_, δ, ppm, 193 K): 102.72, 104.30, 105.20, 106.39, ^1^H NMR (500.15 MHz, THF-*d*_8_, δ, ppm, 193 K): −17.66 (q, μ_4_-H, 1H, ^1^ *J*_1H‑107Ag_ = 27.5 Hz, ^1^ *J*_1H‑109Ag_ = 33.8 Hz). ESI-MS (*m*/*z*): exp. 5017.4326 (calc. for [M + Ag^+^]^+^: 5017.1770). UV–vis [λ_max_ in nm (ε in M^–1^ cm^–1^)]: 395 (57800).

#### Synthesis of [IrHAg_20_{S_2_PPh_2_}_12_] (**1c**)

In a Schlenk tube, [IrHAg_20_{S_2_P­(O^*i*^Pr)_2_}_12_] (0.02 g, 4.2 mmol) was dissolved in THF with 12 equivalents of K­[S_2_PPh_2_] (0.016 g, 50 mmol). The above mixture was stirred at 0 °C for less than 1 min. The reaction mixture was dried under reduced pressure to obtain an orange residue. The residue was extracted with hexane to separate NH_4_[S_2_P­(O^*i*^Pr)_2_]. The hexane solution was dried in vacuo to get an orange powder **1c**. (Yield: 0.02 g, 81.2% based on Ag) **1c**: ^31^P­{^1^H} NMR (202.468 MHz, CDCl_3_, δ, ppm, r.t.): 66.8, ^1^H NMR (500.15 MHz, CDCl_3_, δ, ppm, r.t.): −16.85 (br, μ_4_-H, 1H), 7.38–7.89 (br, 5H). ESI-MS (*m*/*z*): exp. 6143.8604 (calc. for [M + Ag]^+^: 6143.8701). UV–vis [λ_max_ in nm (ε in M^–1^ cm^–1^)]: 382 (46000), 436 (30000).

#### Synthesis of [IrHAg_20_{Se_2_P­(O^*i*^Pr)_2_}_12_] (**2**)

An analogous procedure to **1c** except for the substitution of the ligand for NH_4_[Se_2_P­(O^*n*^Pr)_2_] (0.016 g, 50 mmol). **2** (Yield: 0.02 g, 79.8%, based on Ag): ^31^P­{^1^H} NMR (202.468 MHz, CDCl_3_, δ, ppm, r.t.): 66.8, ^1^H NMR (500.15 MHz, CDCl_3_, δ, ppm, r.t.): −16.85 (br, H, H), 1.37 (br, CH_3_, 144H), 4.96 (br, OCH, 24H). ESI-MS (*m*/*z*): exp. 6143.8604 (calc. for [M + Ag]^+^: 6143.8701). UV–vis [λ_max_ in nm (ε in M^–1^ cm^–1^)]: 385 (36000), 433 (24000).

### X-ray Crystallography

Single crystals suitable for X-ray diffraction analysis of **1a**, **1b**, and **2** were obtained by the slow evaporation of a MeOH solution at 4 °C for 1 week. Single crystals suitable for X-ray diffraction analysis of **1c** were obtained by the slow evaporation of a DCM solution at 4 °C for 1 week. The single crystals were mounted on the tip of a glass fiber coated in paratone oil, then frozen. Data were collected on a Bruker APEX II CCD diffractometer using graphite monochromated Mo *K*α radiation (λ = 0.71073 Å) at 100 K. Absorption corrections for the area detector were performed with SADABS,^40^ and the integration of raw data frames was performed with SAINT.^41^ The structure was solved by direct methods and refined by least-squares against *F* ^2^ using the SHELXL-2018/3 package,^42,43^ incorporated in SHELXTL/PC V6.14.^44^ All non-hydrogen atoms were refined anisotropically.

### Electrochemical Study

All electrochemical data were collected on a Squidstat Plus potentiostat (Admiral Instruments). Compounds **1a**, **1b**, **1c**, and **2** were dissolved in the solution containing 0.1 M [TBA]­[PF_6_] DCM solution and cooled to 253 K to improve the electrochemical stability window of both the cluster and the electrolyte. The chamber and solution were purged with argon gas for 10 min before each scan. A three-electrode system consiting of a glassy-carbon working electrode, Ag/AgCl reference electrode, and Pt wire as a counter electrode was utilized for all measurements.

### Computational Details

Geometry optimizations were performed within the formalism of density functional theory (DFT), with the Gaussian 16 package,^45^ using the BP86 functional^46^ and the all-electron Def2-TZVP set from EMSL Basis Set Exchange Library.^47^ All of the optimized geometries were characterized as true minima by vibrational analysis. The natural population analysis (NPA) and Wiberg bond indices were computed with the NBO 6.0 program.^48^ The UV–visible transitions were calculated by means of time-dependent DFT (TD-DFT) calculations, with the CAM-B3LYP functional^49^ and the Def2-TZVP basis set. Only singlet–singlet, *i*.*e*., spin-allowed, transitions have been computed. The UV–visible spectra were simulated from the computed TD-DFT transitions and their oscillator strengths by using the Multiwfn program,^50^ and each transition is associated with a Gaussian function of half-height width equal to 1000 cm^–1^. The compositions of the molecular orbitals were calculated using the AOMix program.^51^

## Supplementary Material



## Notes

Additional experimental details, methods, spectra (^1^H/^31^P NMR, XPS, ESI-MS, electrochemical), and computational data (Kohn–Sham frontier orbitals, TD-DFT simulated UV–vis spectra) (PDF)
