# Supplementary material for: Ligand-Induced Structural Evolution and Luminescence Tuning in a Series of Superatomic Ir/Ag Hydride-Containing Nanoclusters
Source: Inorg Chem. 2026 Jun 19;65(26):14839–47. doi: 10.1021/acs.inorgchem.6c01668 (PMC13343461; doi:10.1021/acs.inorgchem.6c01668)
Supplement: Supplementary file 1 [file ic6c01668_si_001.pdf]

Supporting Information

Ligand-induced structural evolution and luminescence tuning in a series of superatomic Ir/Ag hydride-containing nanoclusters

Wei-Jung Yen,[a] Tzu-Hao Chiu,[a] Michael N. Pillay,[a] Samia Kahlal,[b] Jean-Yves Saillard,[b*] and C. W. Liu[a*]

[a] Department of Chemistry, National Dong Hwa University, Hualien 97401, Taiwan (Republic of China), chenwei@gms.ndhu.edu.tw

[b] Univ Rennes, CNRS, ISCR-UMR 6226, F-35000 Rennes, France, jean-yves.saillard@univ-rennes1.fr

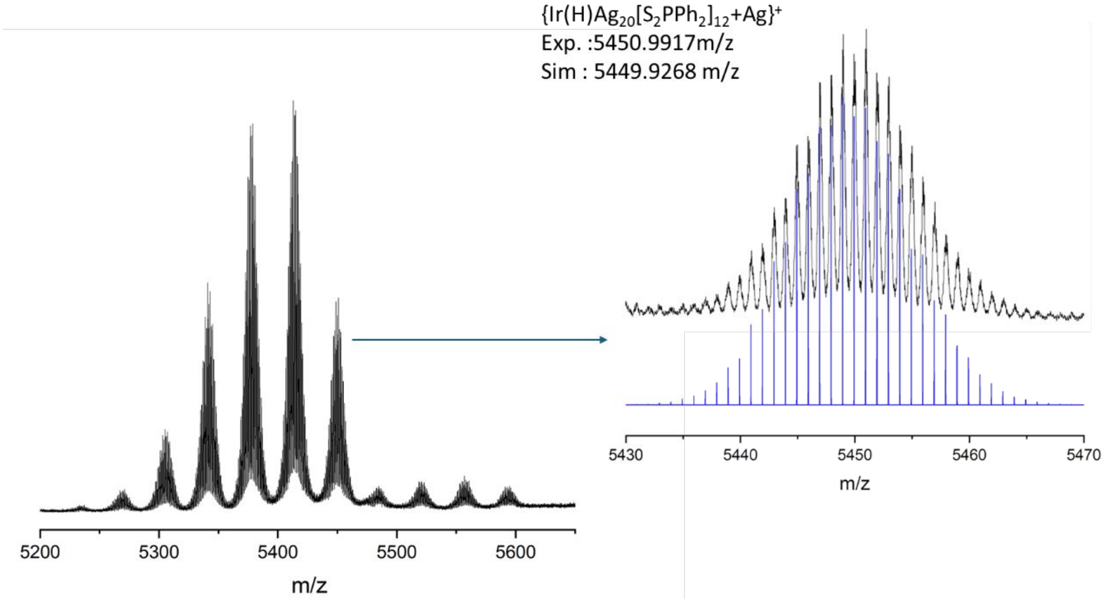

Figure S1. The ESI-Mass spectrum of **1c** the molecular ion peak corresponding to [**1c**+Ag]$^+$

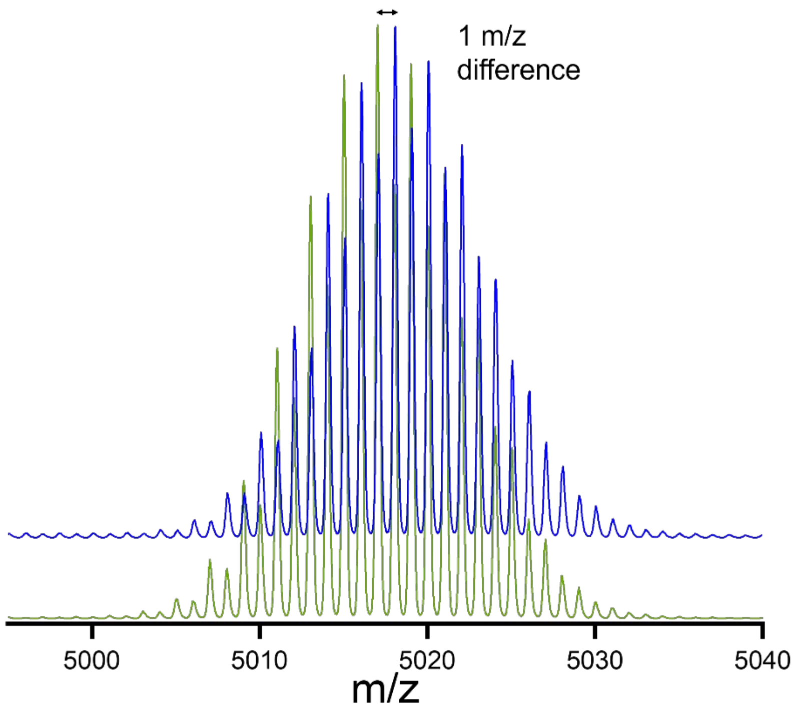

Figure S2. The ESI-Mass spectrum of the **1a** and **1a-D** the molecular ion peak corresponding to the [**1a+Ag**] (green) and [**1a-D+Ag**] (blue).

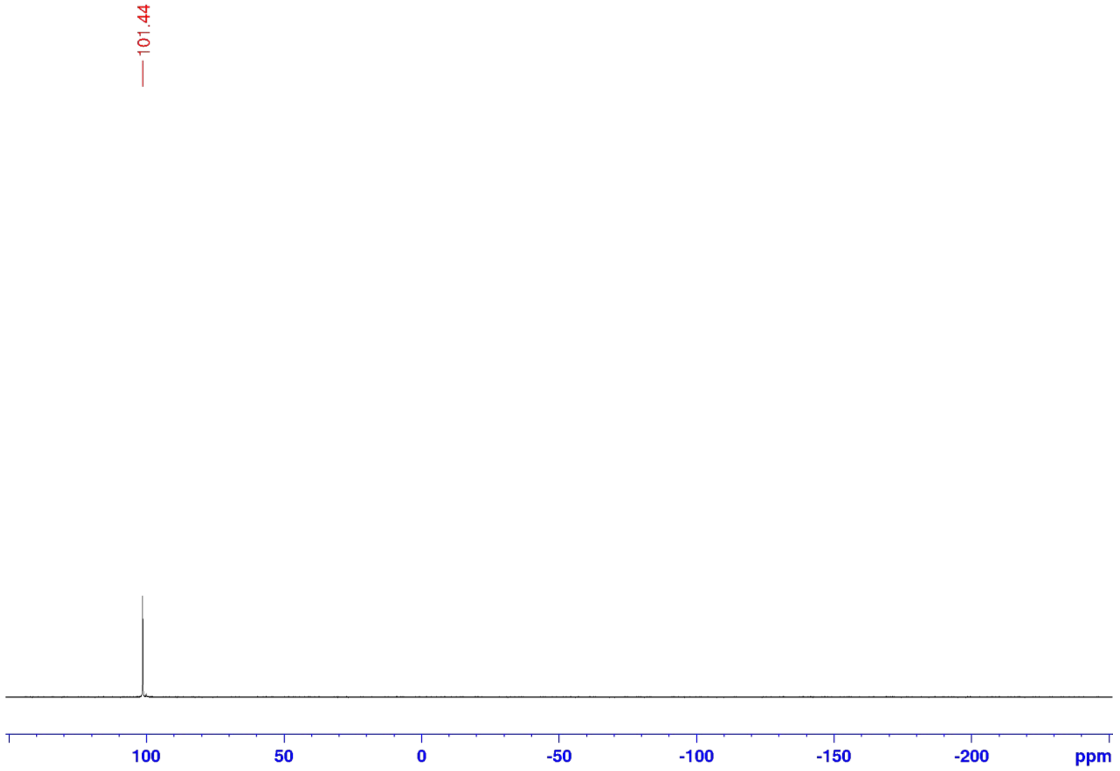

Figure S3. ^{31}P NMR spectrum of **1a** in $CDCl_3$.

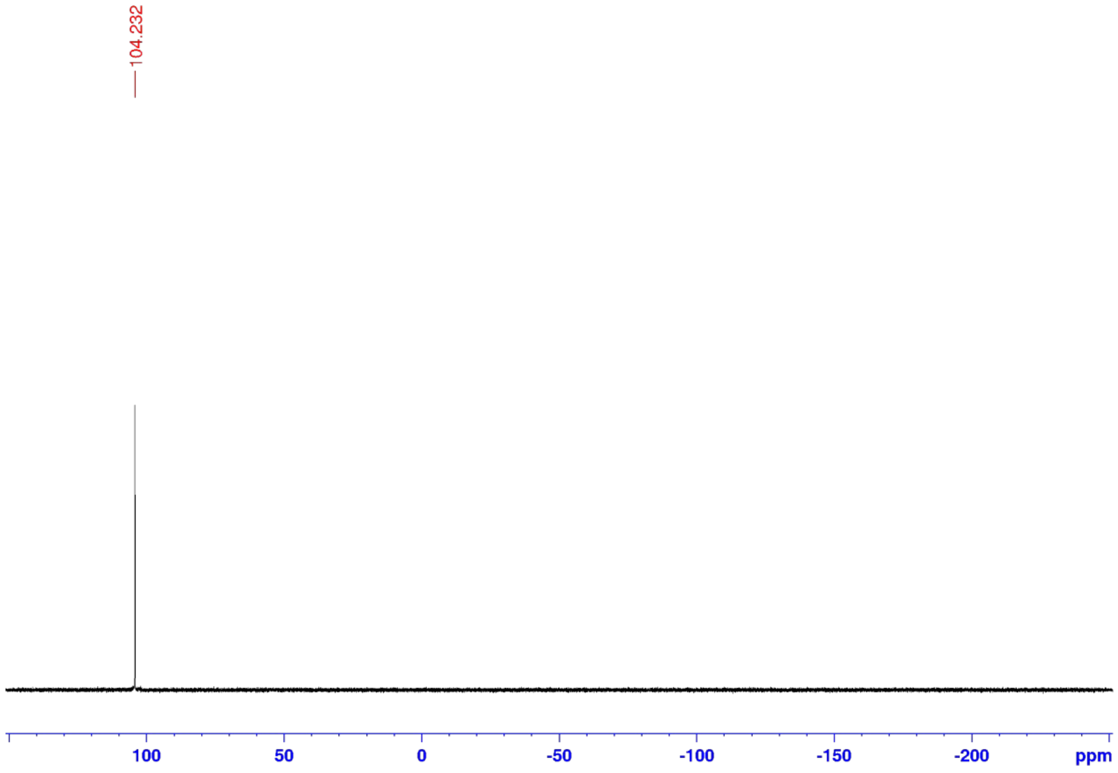

Figure S4. ^{31}P NMR spectrum of **1b** in $CDCl_3$.

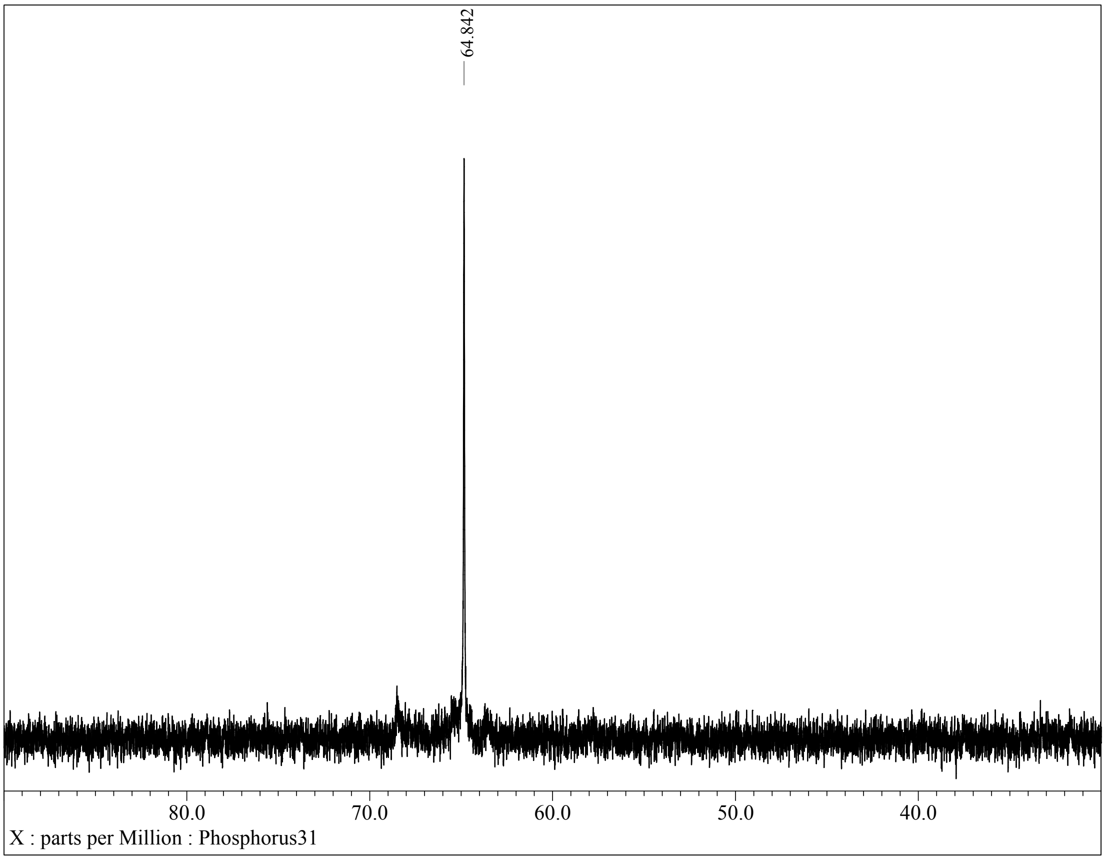

Figure S5. ^{31}P NMR spectrum of **1c** in *d8*-THF.

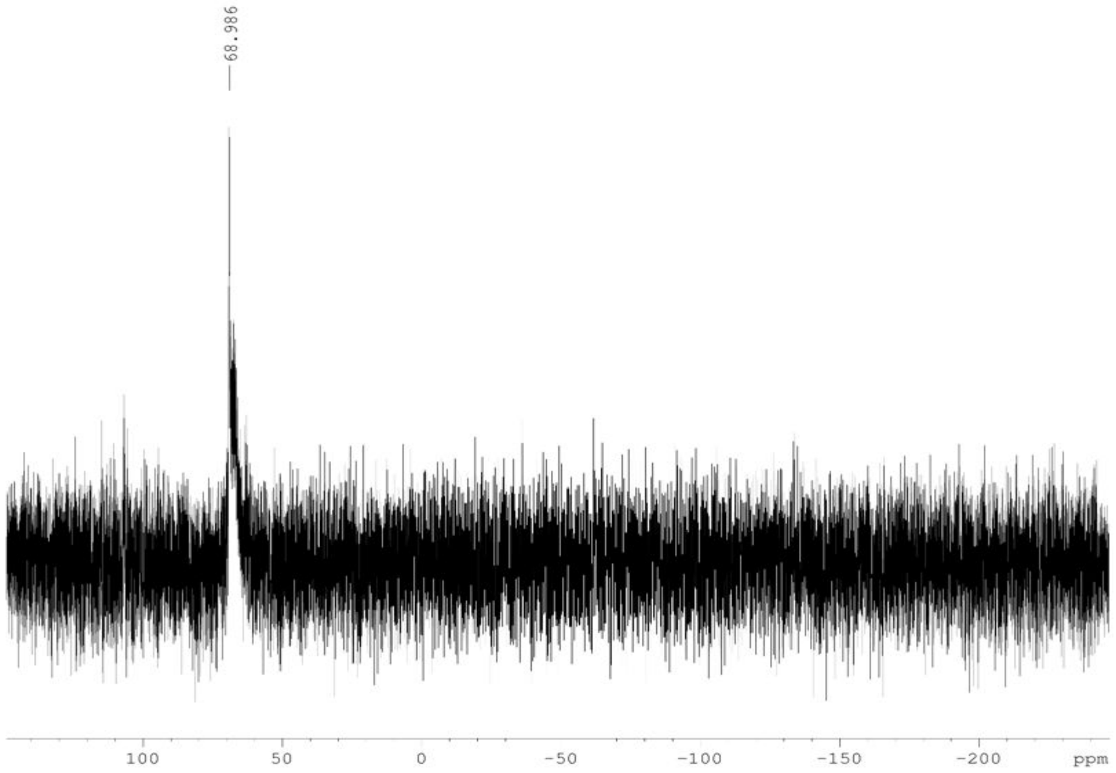

Figure S6. ^{31}P NMR spectrum of **2** in $CDCl_3$.

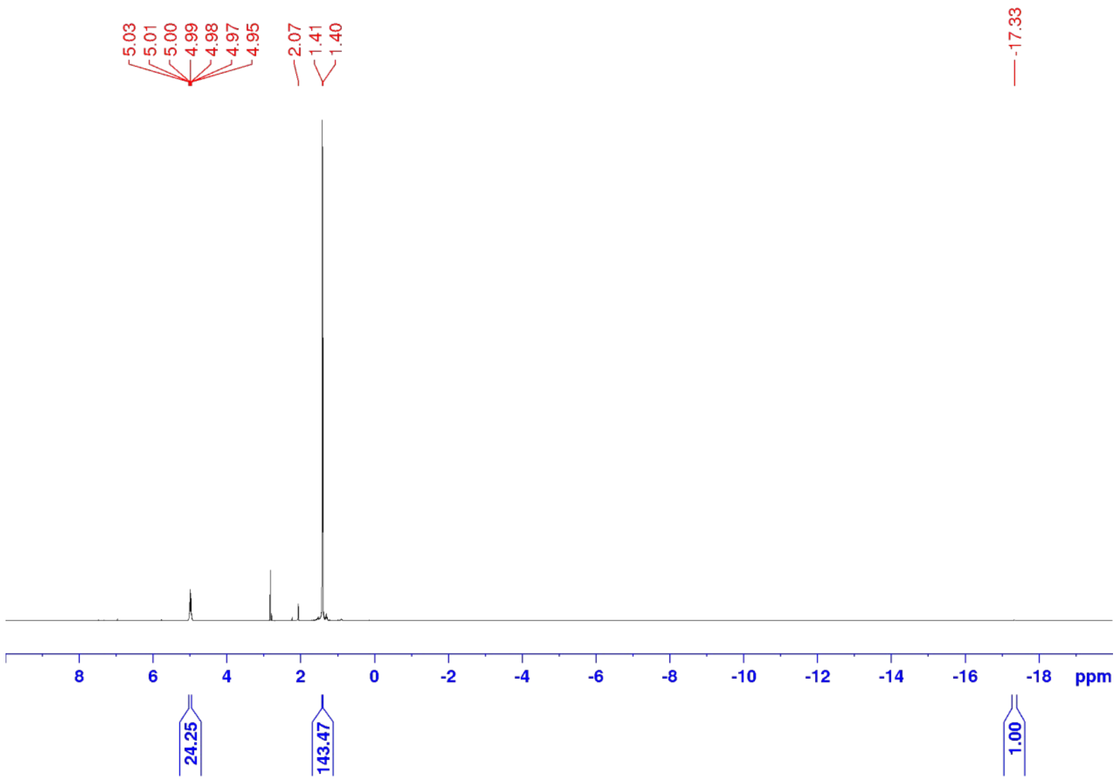

Figure S7. ^{1}H NMR spectrum of **1a** in $CDCl_3$.

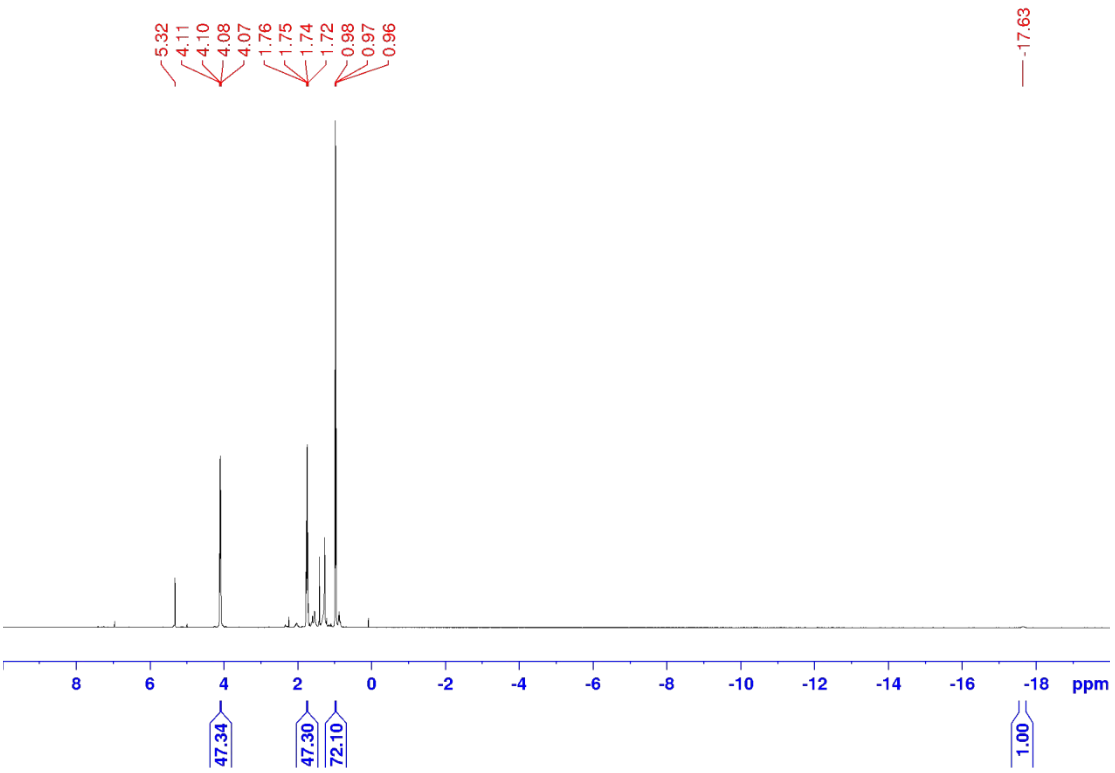

Figure S8. ^{1}H NMR spectrum of **1b** in $CDCl_3$.

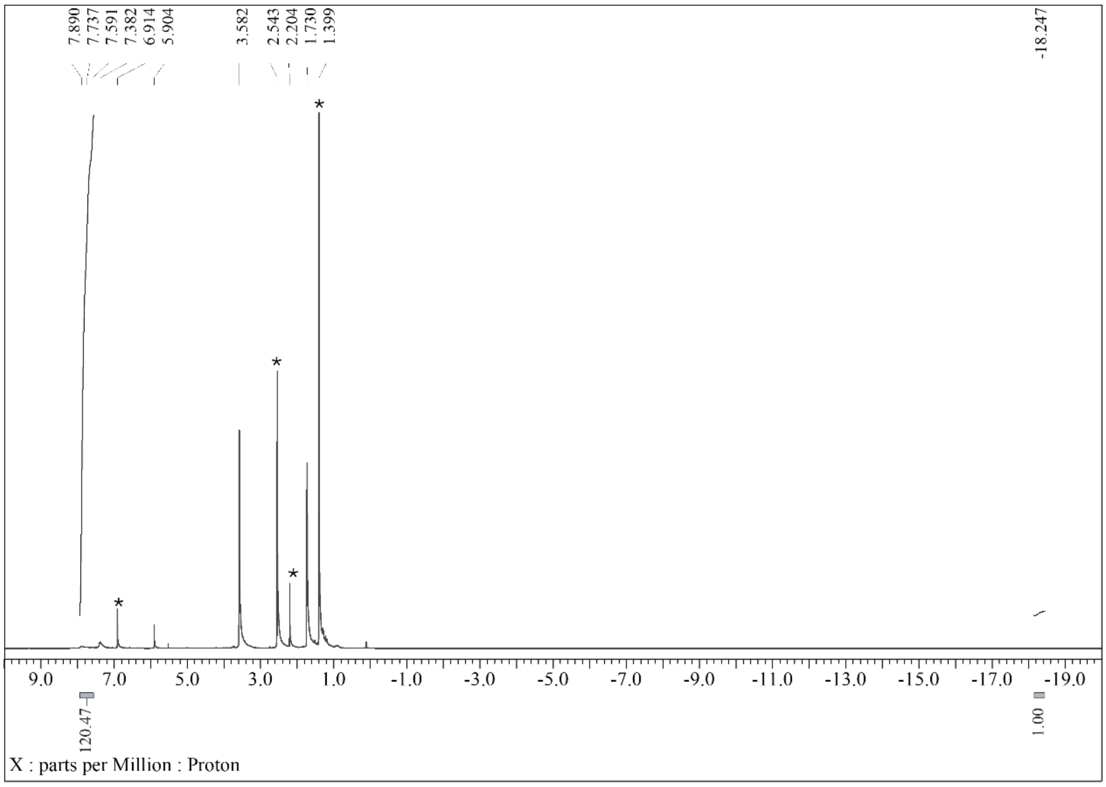

Figure S9. ^{1}H NMR spectrum of **1c** in *d8*-THF. (* is the BHT chemical shift)

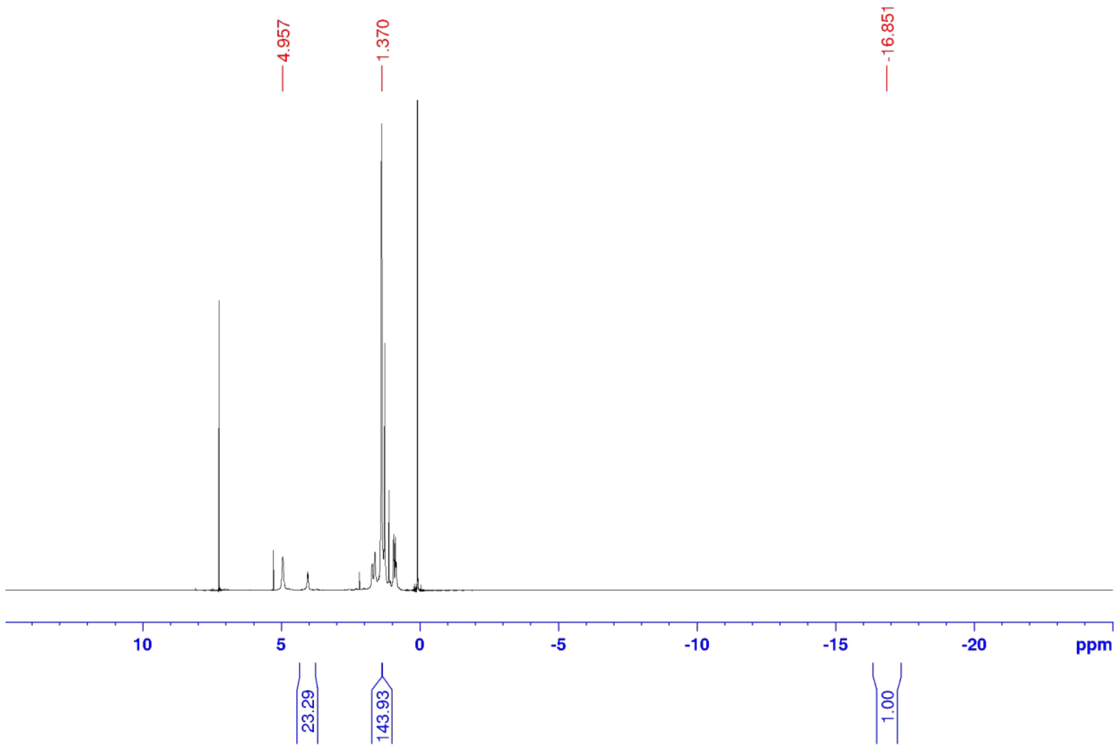

Figure S10. ^{1}H NMR spectrum of **2** in $CDCl_3$

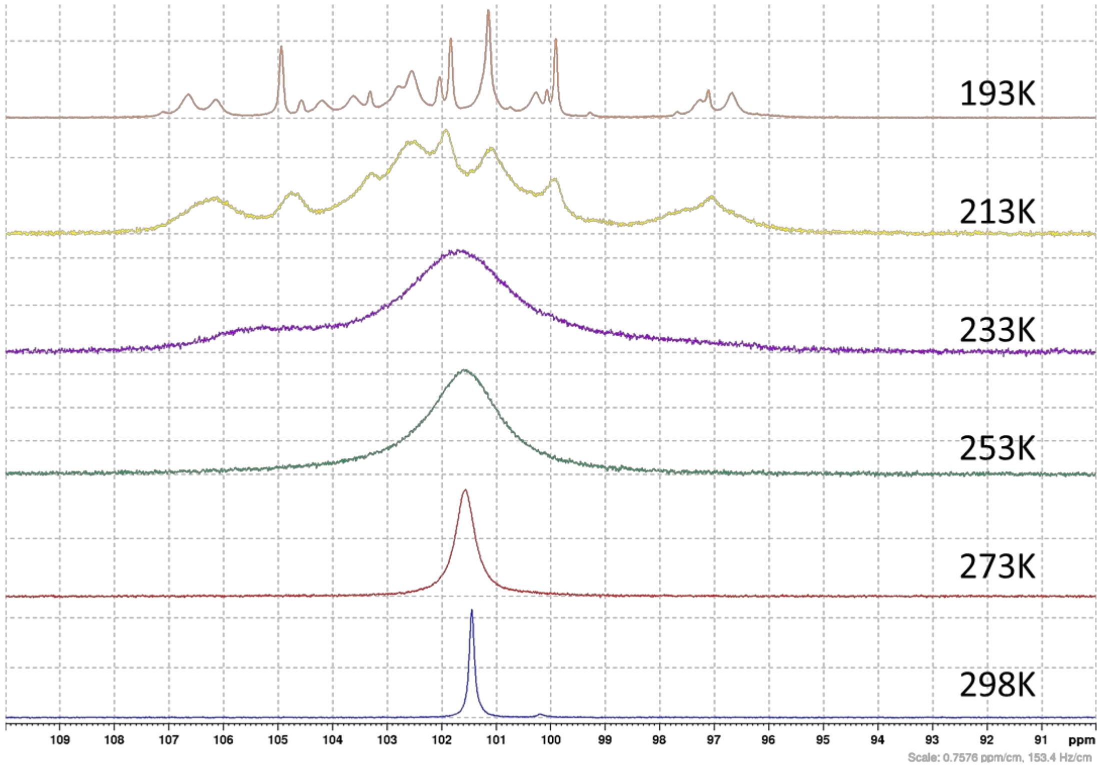

Figure S11. VT ^{31}P NMR spectrum of **1a** in *d8*-THF.

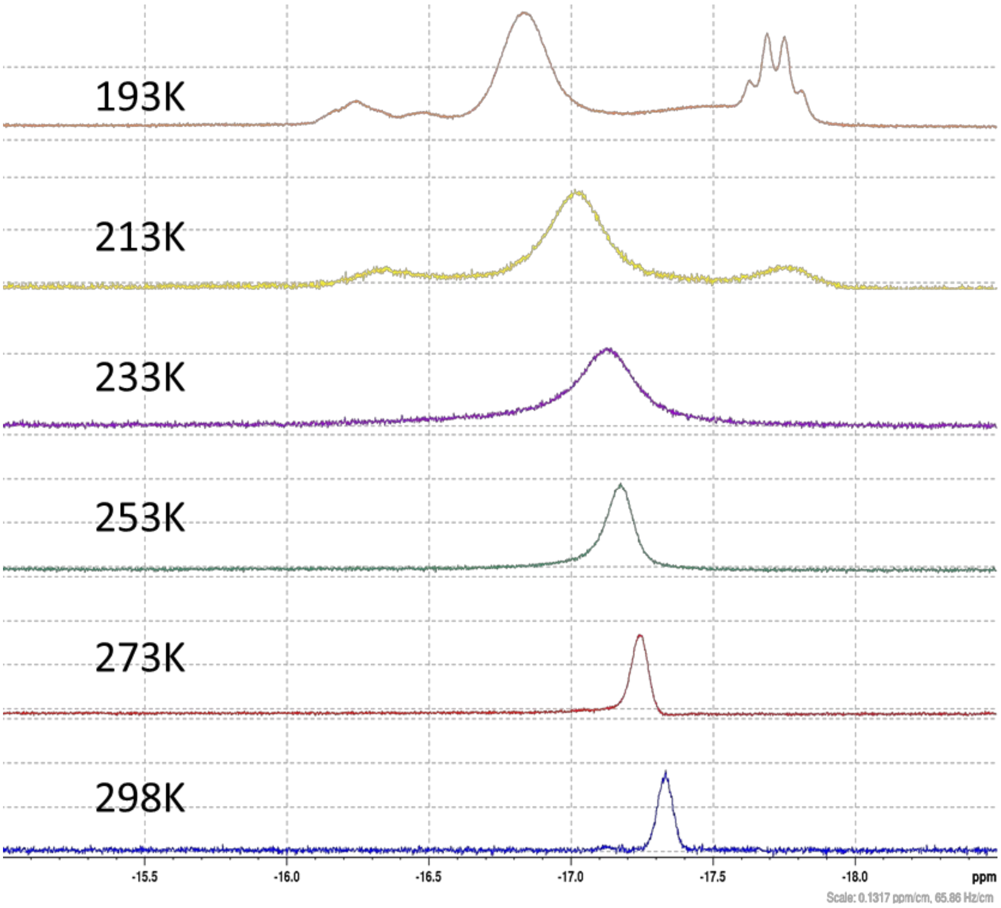

Figure S12. VT ^{1}H NMR spectrum of **1a** in *d8*-THF.

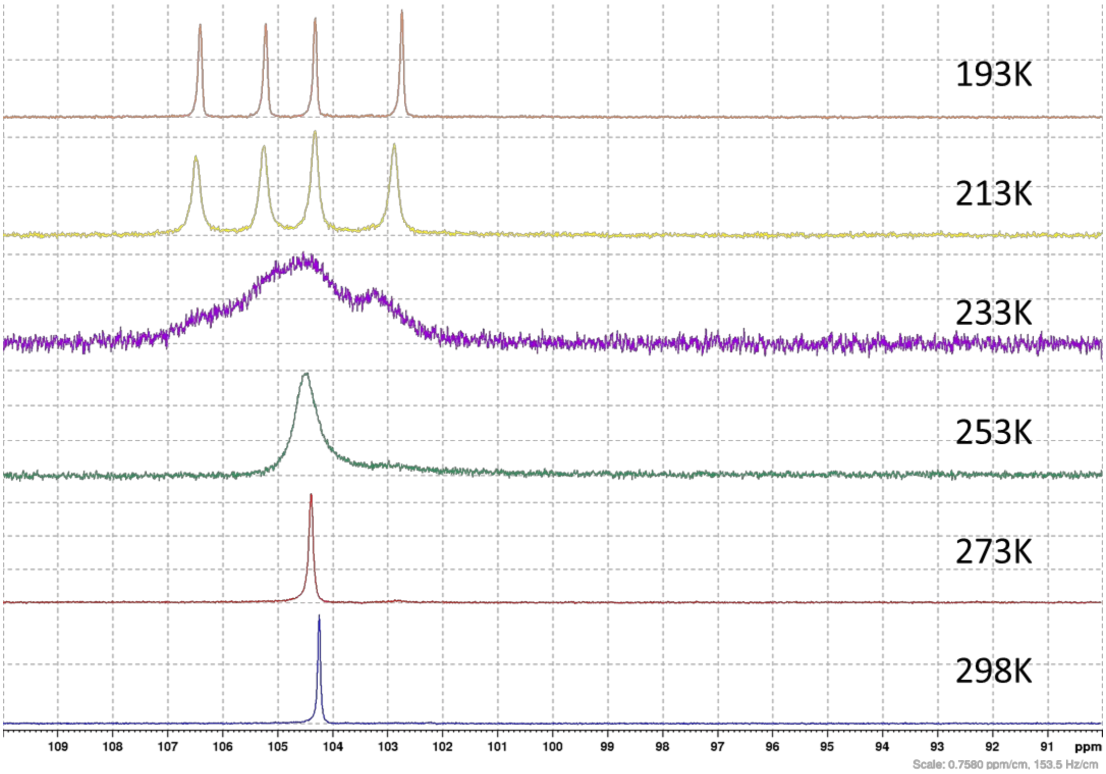

Figure S13. VT ^{31}P NMR spectrum of **1b** in *d8*-THF.

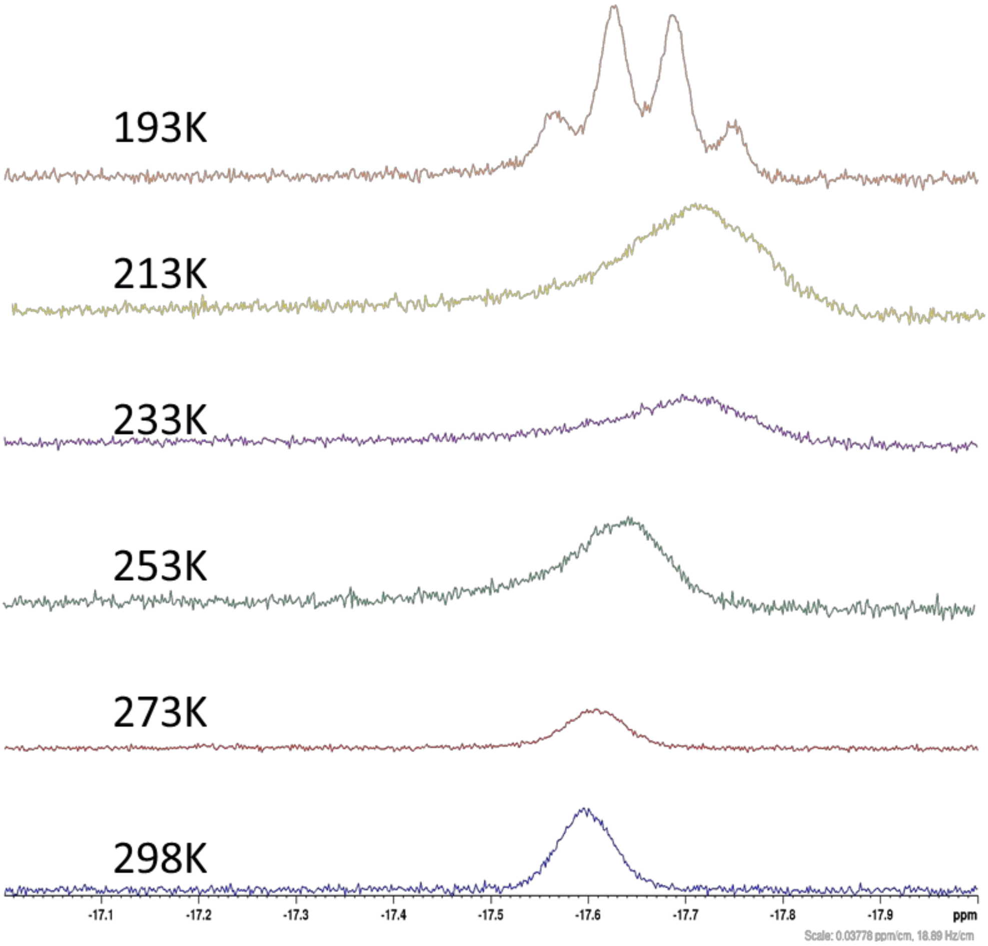

Figure S14. VT ^{1}H NMR spectrum of **1b** in *d8*-THF.

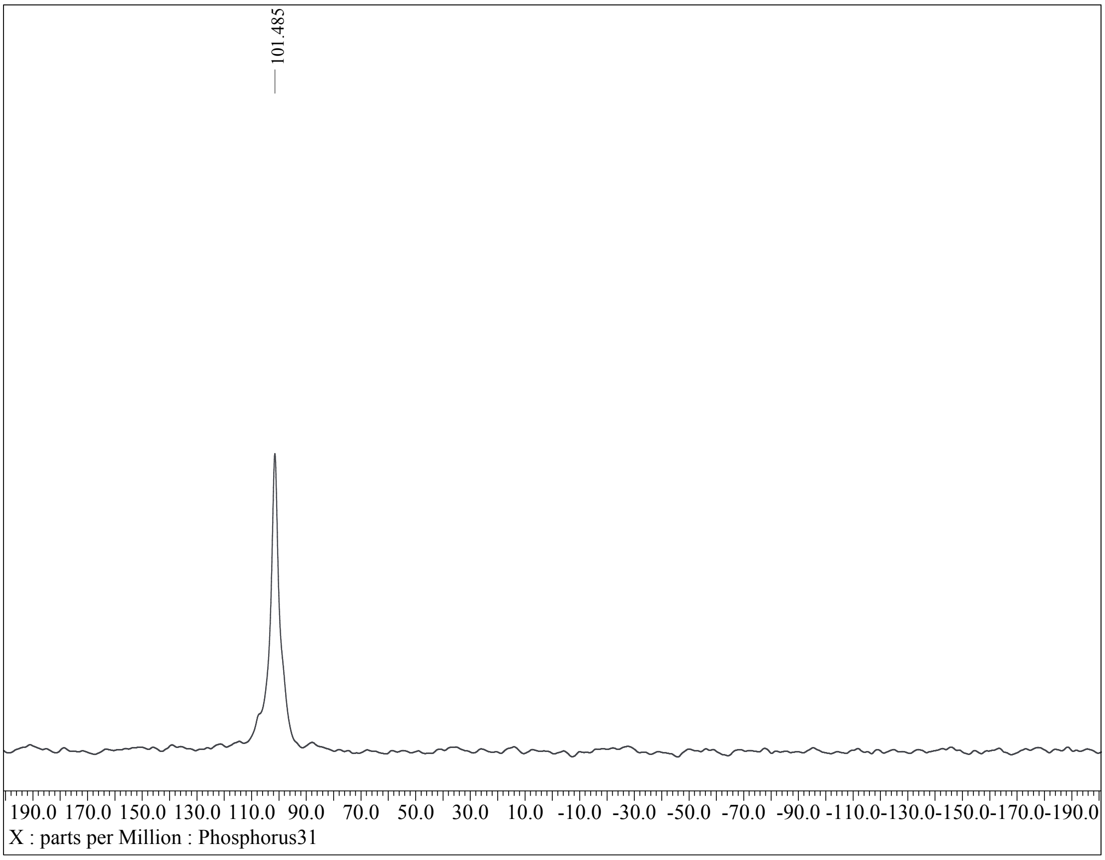

Figure S15. ^{31}P NMR spectrum of **1a-D** in CH_2Cl_2.

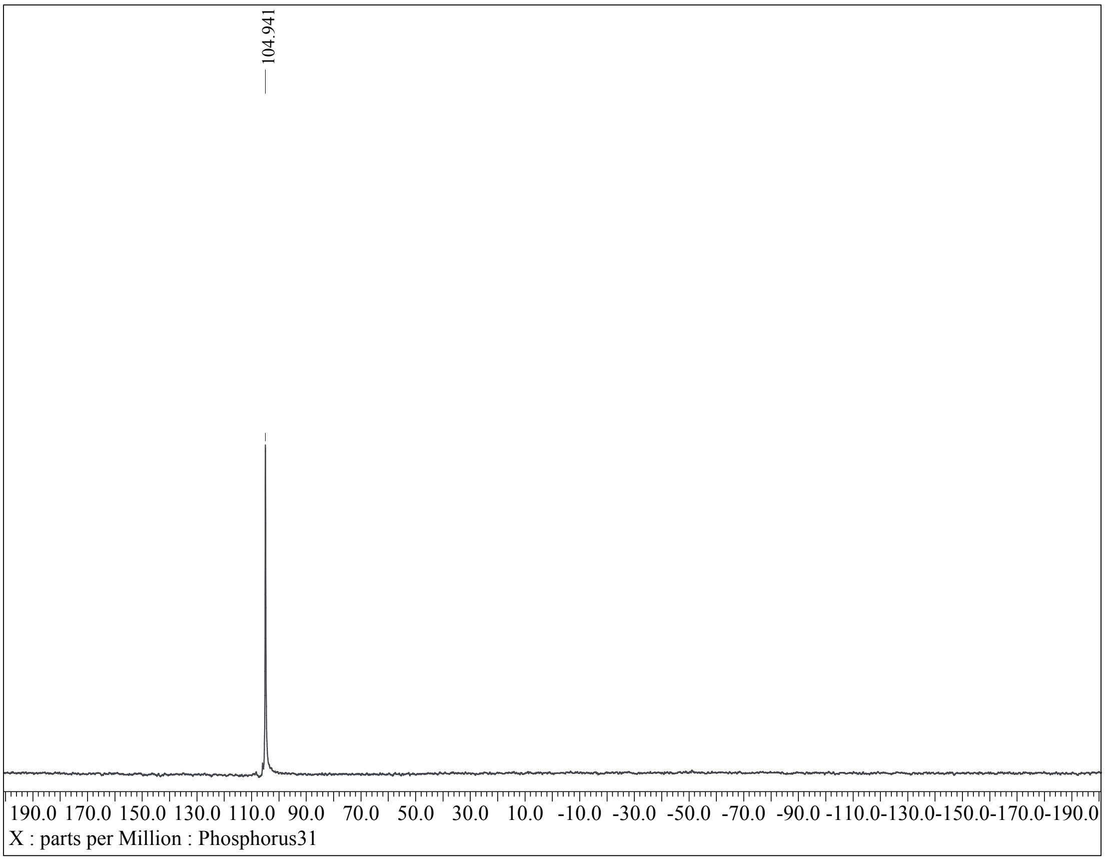

Figure S16. ^{31}P NMR spectrum of **1b-D** in CH_2Cl_2.

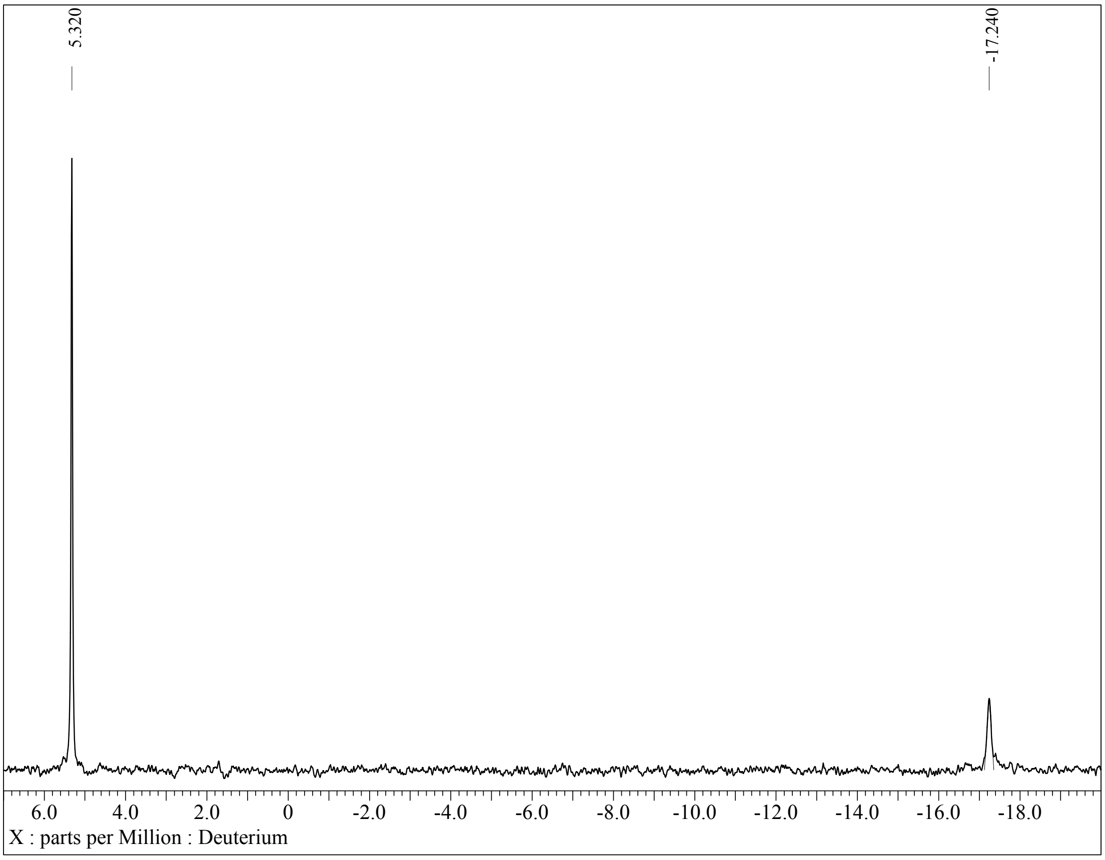

Figure S17. ^{2}H NMR spectrum of **1a-D** in CH_2Cl_2.

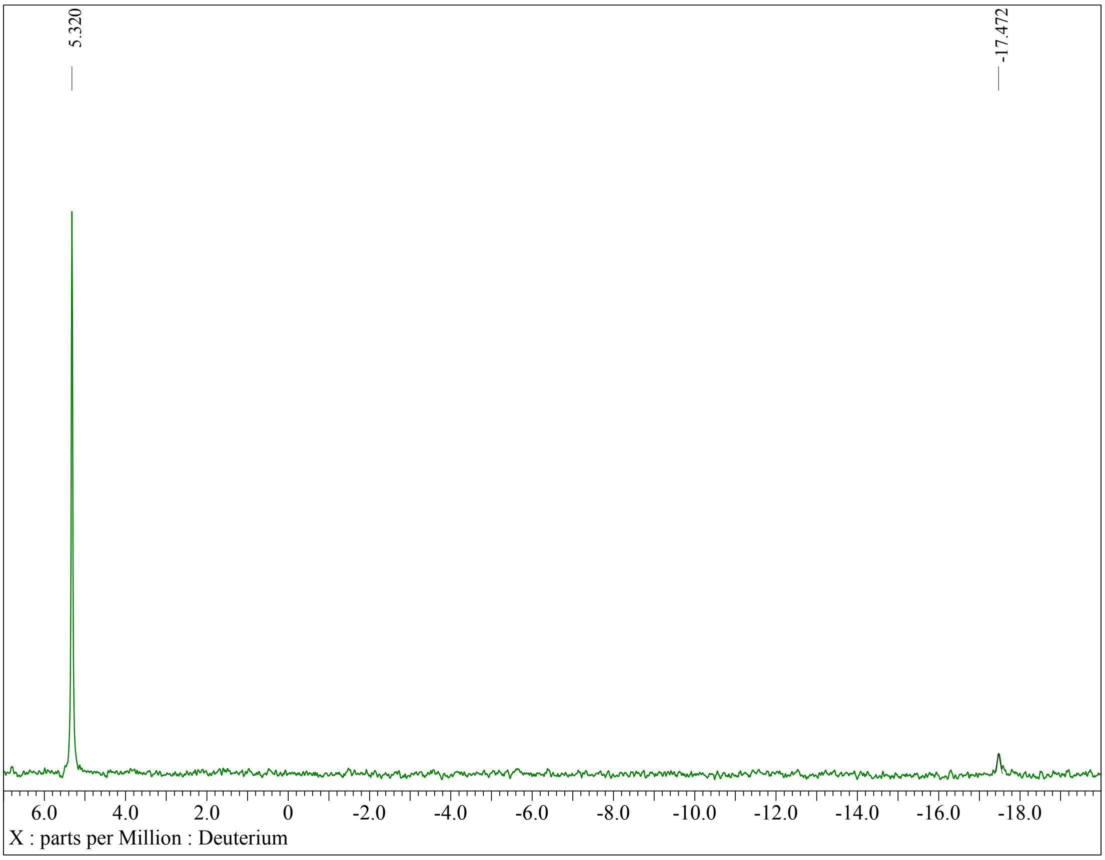

Figure S18. ^{2}H NMR spectrum of **1b-D** in CH_2Cl_2.

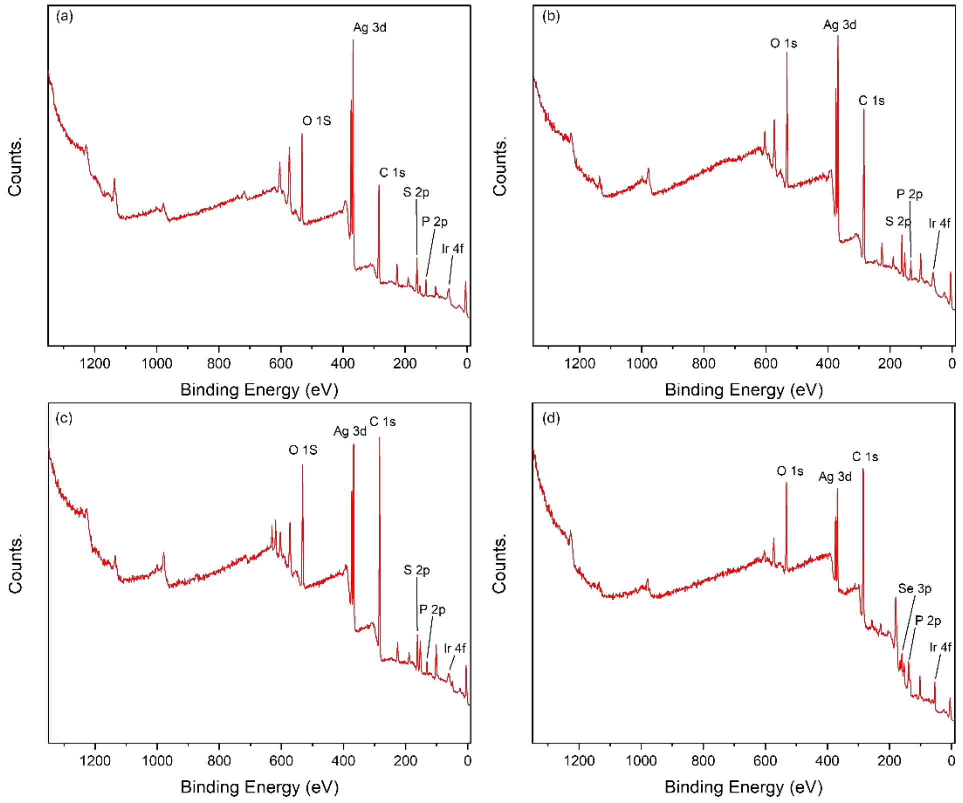

Figure S19. The XPS spectrum full map of (a) **1a**, (b) **1b**, (c) **1c,** and (d) **2**.

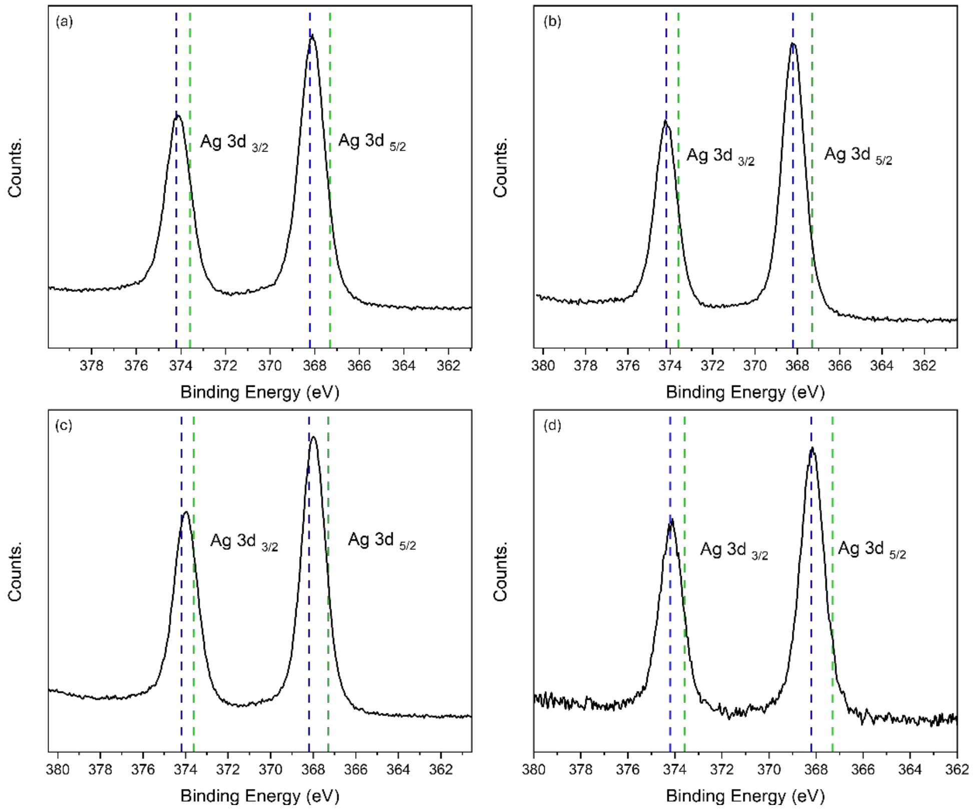

Figure S20. The XPS spectrum of Ag $3d_{5/2}$ and Ag $3d_{3/2}$ (a) **1a**, (b) **1b**, (c) **1c,** and (d) **2**. The dashed line indicates the standard binding energy of the Ag(0) (Blue) and Ag(I)

(Green).

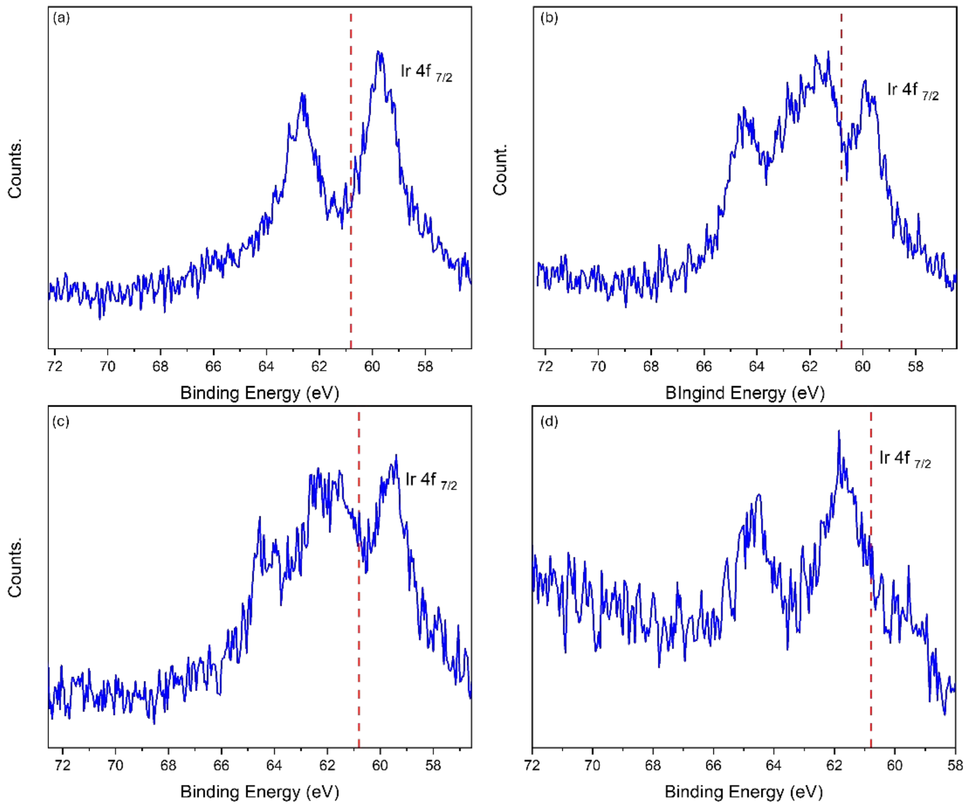

Figure S21. The XPS spectrum of Ir 4f $_{7/2}$ (a) **1a**, (b) **1b**, (c) **1c,** and (d) **2**. The dashed line indicates the standard binding energy of the Ir(0).

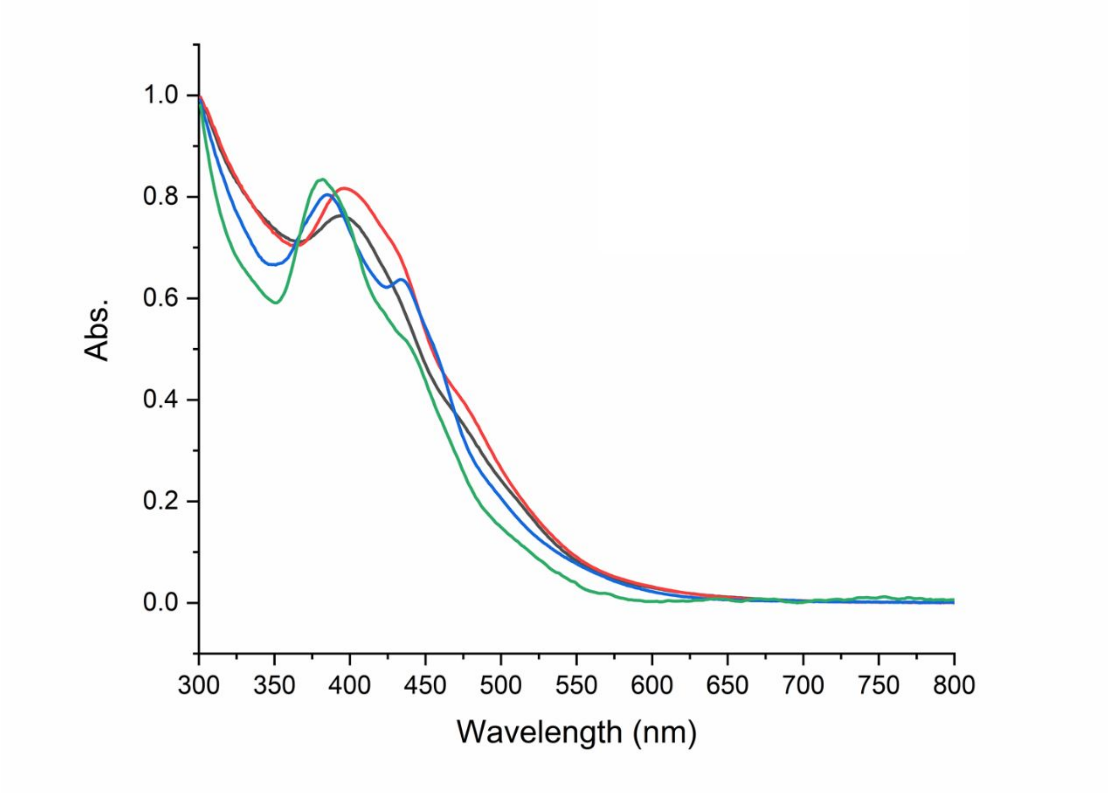

Figure S22. UV-vis absorption spectra of **1-2** (red: **1a**; black: **1b**; green: **1c**; blue: **2**)

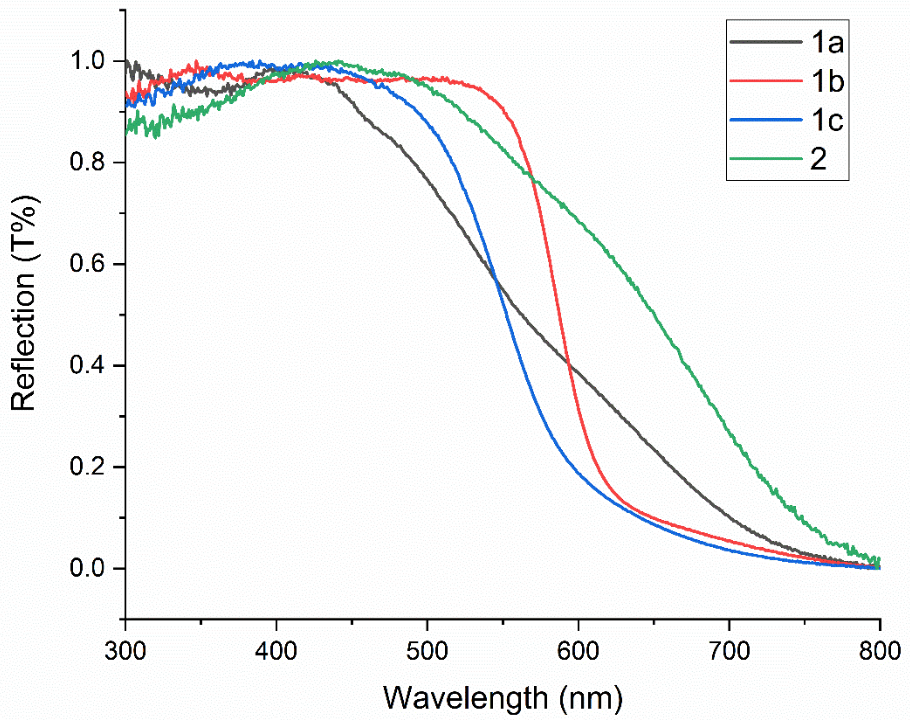

Figure S23. Diffuse reflection spectra of **1-2**.

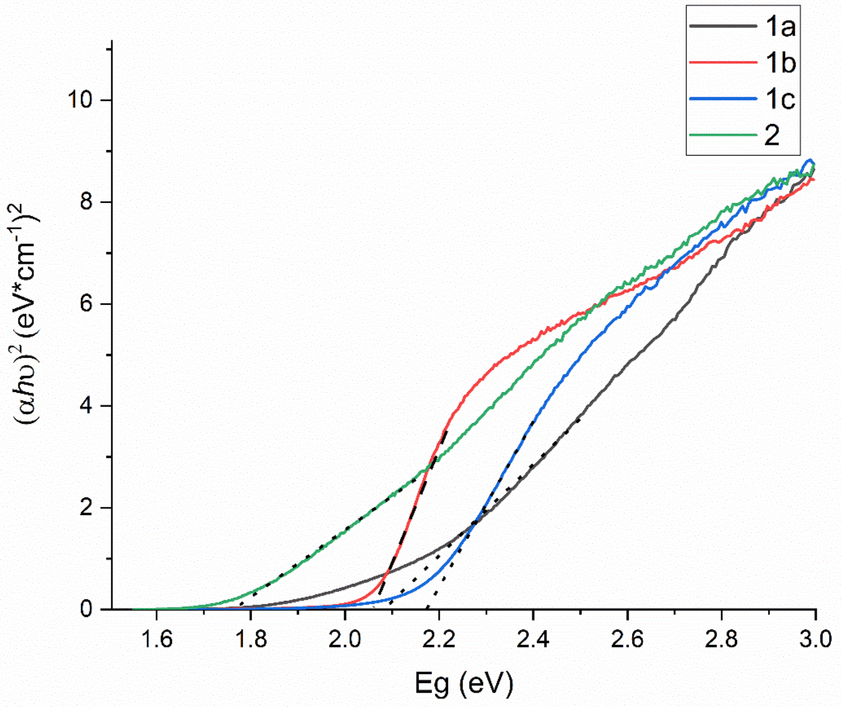

Figure S24. Tauc plot of **1-2**. (Eg: **1a** = 2.08 eV, **1b** = 2.06 eV, **1c** = 2.17 eV, **2** = 1.77 eV)

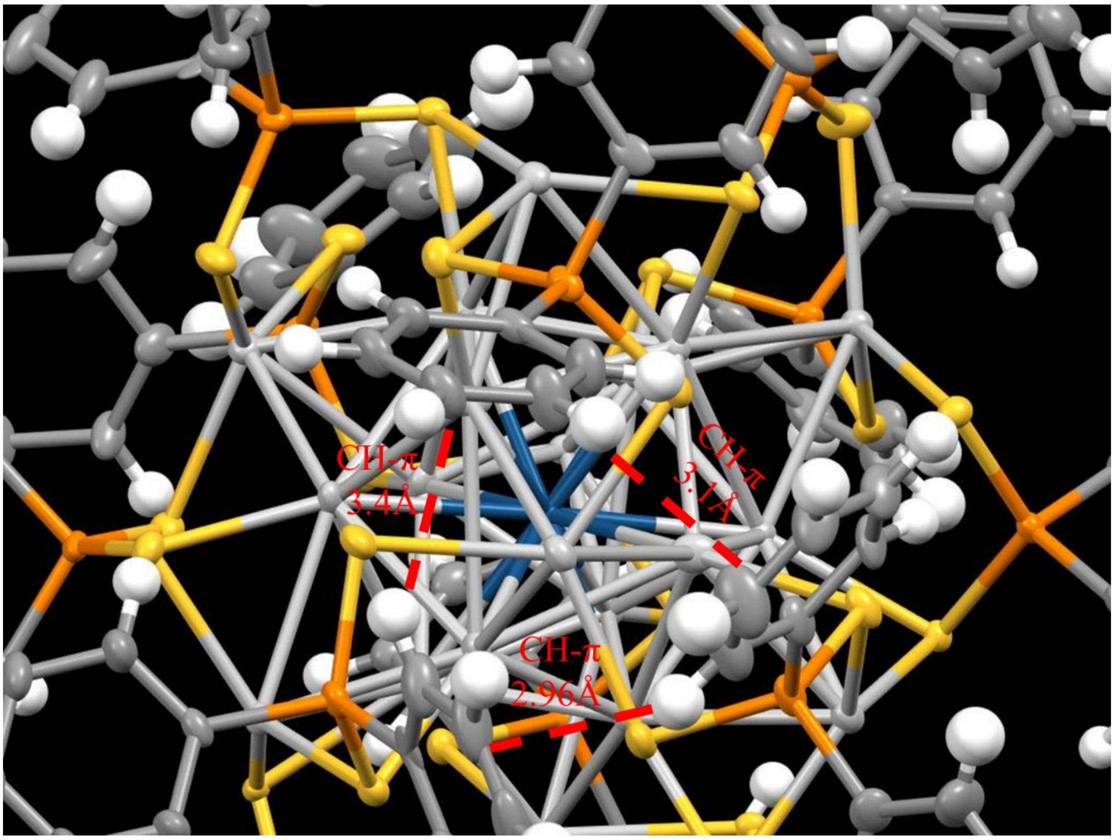

Figure S25. Intramolecular C–H to π interactions

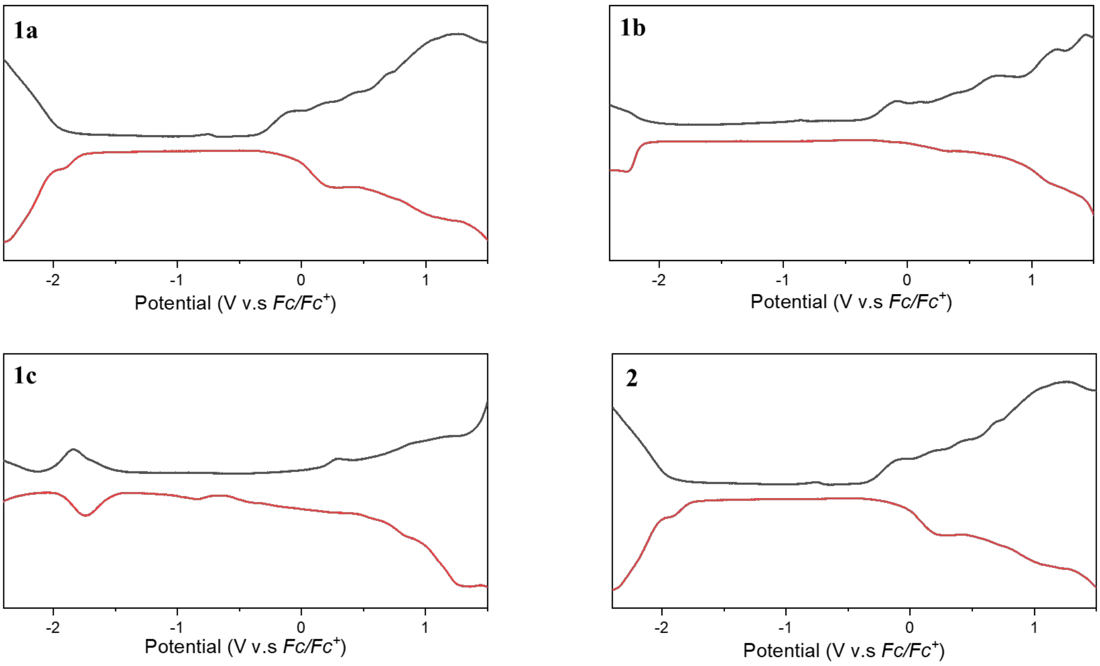

Figure S26. Differential pulse voltammograms of **1a**, **1b**, **1c** and **2**

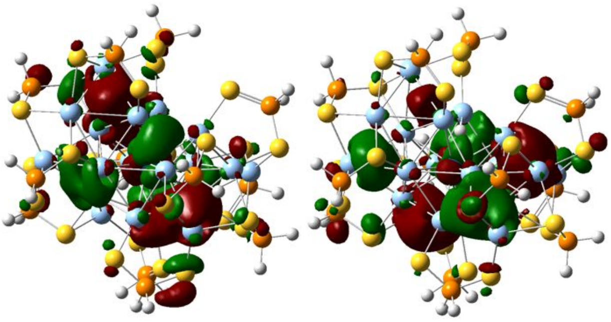

1a_LUMO+3 7/0/56/19/18 | 1a_LUMO+4 2/0/54/24/19

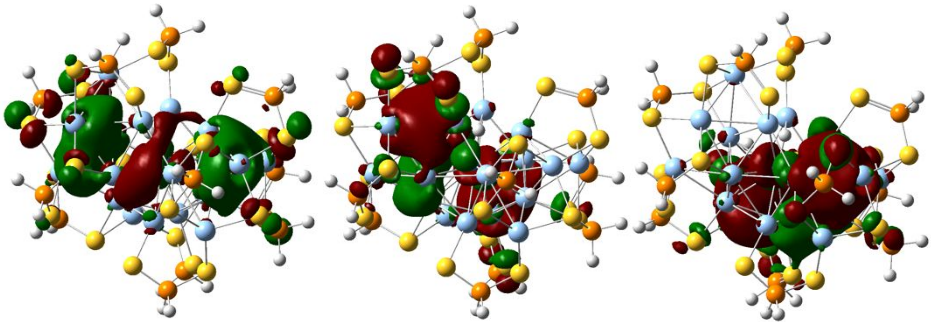

1a_LUMO 12/1/44/27/17 | 1a_LUMO+1 9/0/50/24/17 | 1a_LUMO+2 8/0/52/22/18

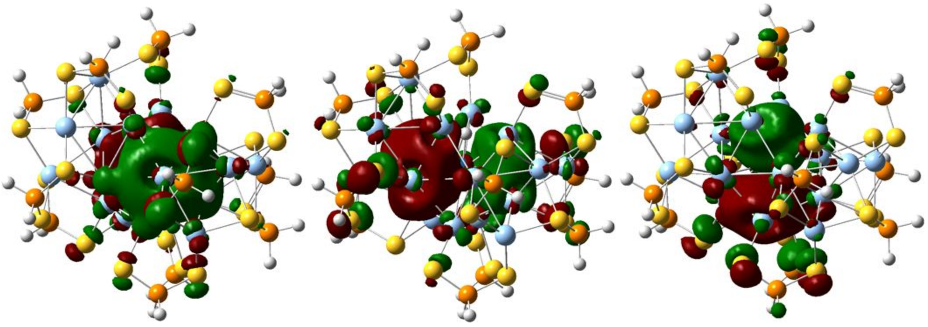

1a_HOMO 24/0/47/7/23 | 1a_HOMO-1 25/0/41/9/25 | 1a_HOMO-2 39/3/36/2/20

Figure S27. Kohn-Sham frontier orbitals of **1a**. Atomic contributions (in %) are given in the order: Ir/H/$Ag_{icosahedron}$/$Ag_{capping}$/ligands.

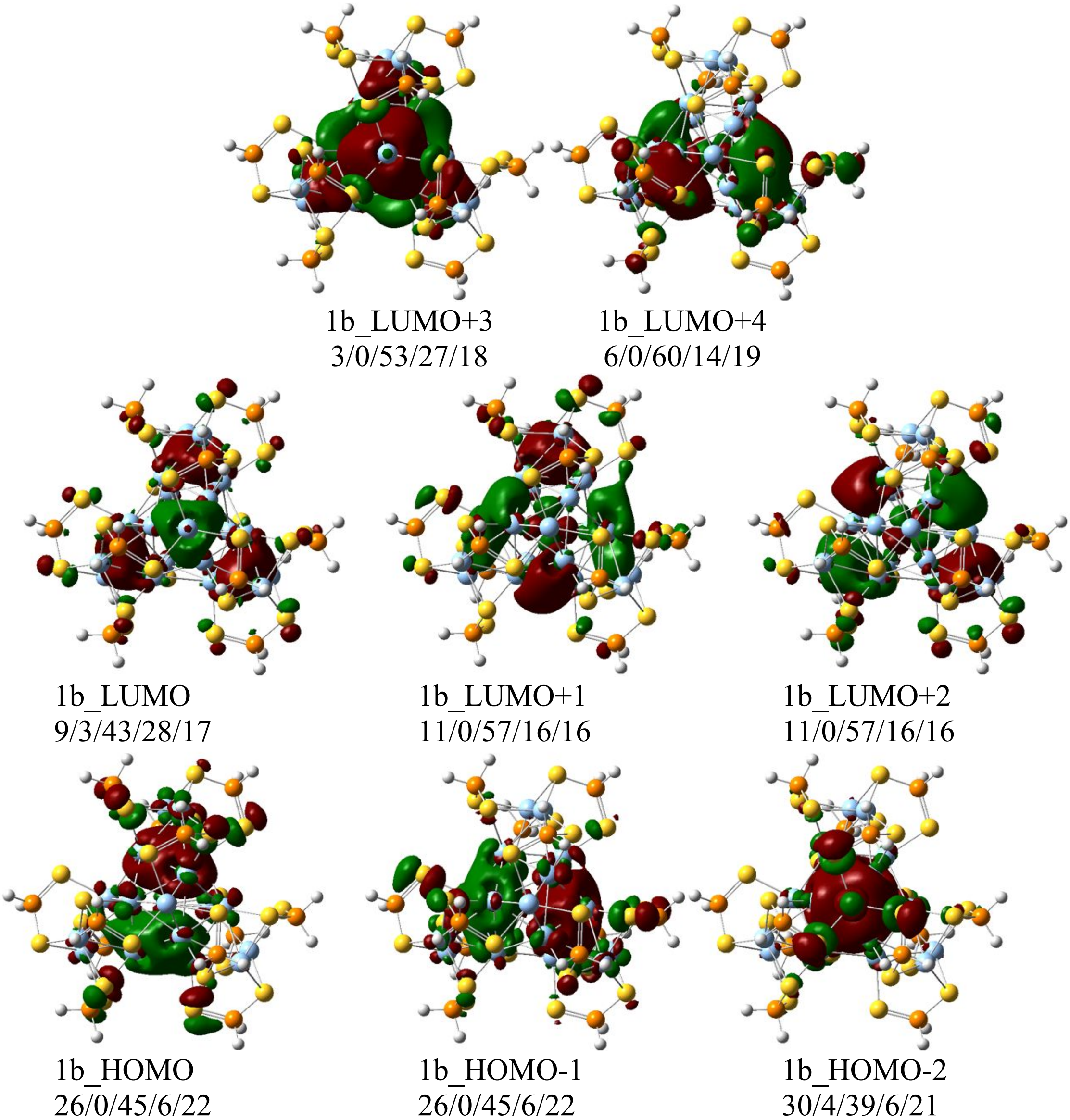

Figure S28. Kohn-Sham frontier orbitals of **1b**. Atomic contributions (in %) are given in the order: Ir/H/$Ag_{icosahedron}$/$Ag_{capping}$/ligands.

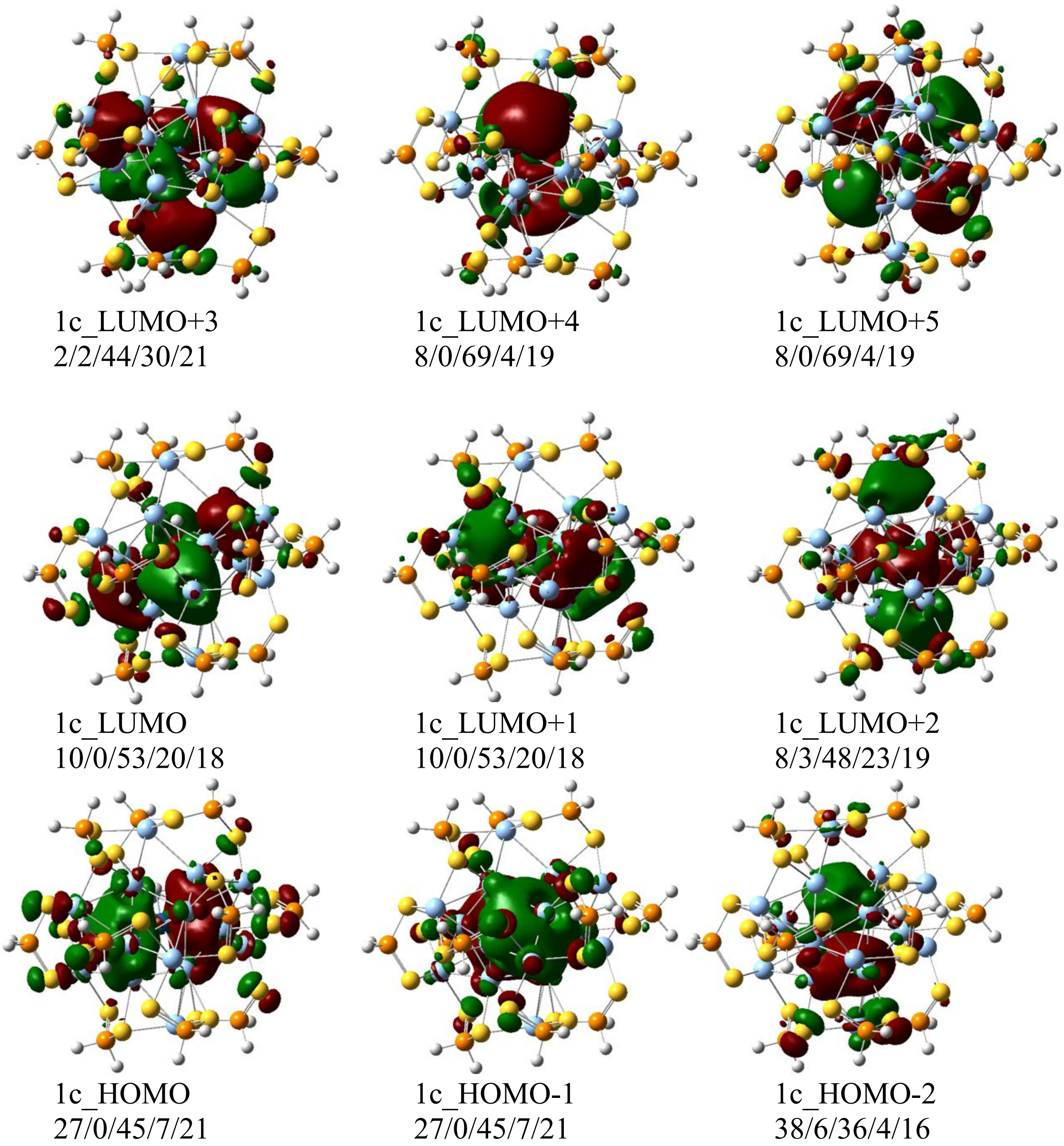

Figure S29. Kohn-Sham frontier orbitals of **1c**. Atomic contributions (in %) are given in the order: Ir/H/$Ag_{icosahedron}$/$Ag_{capping}$/ligands.

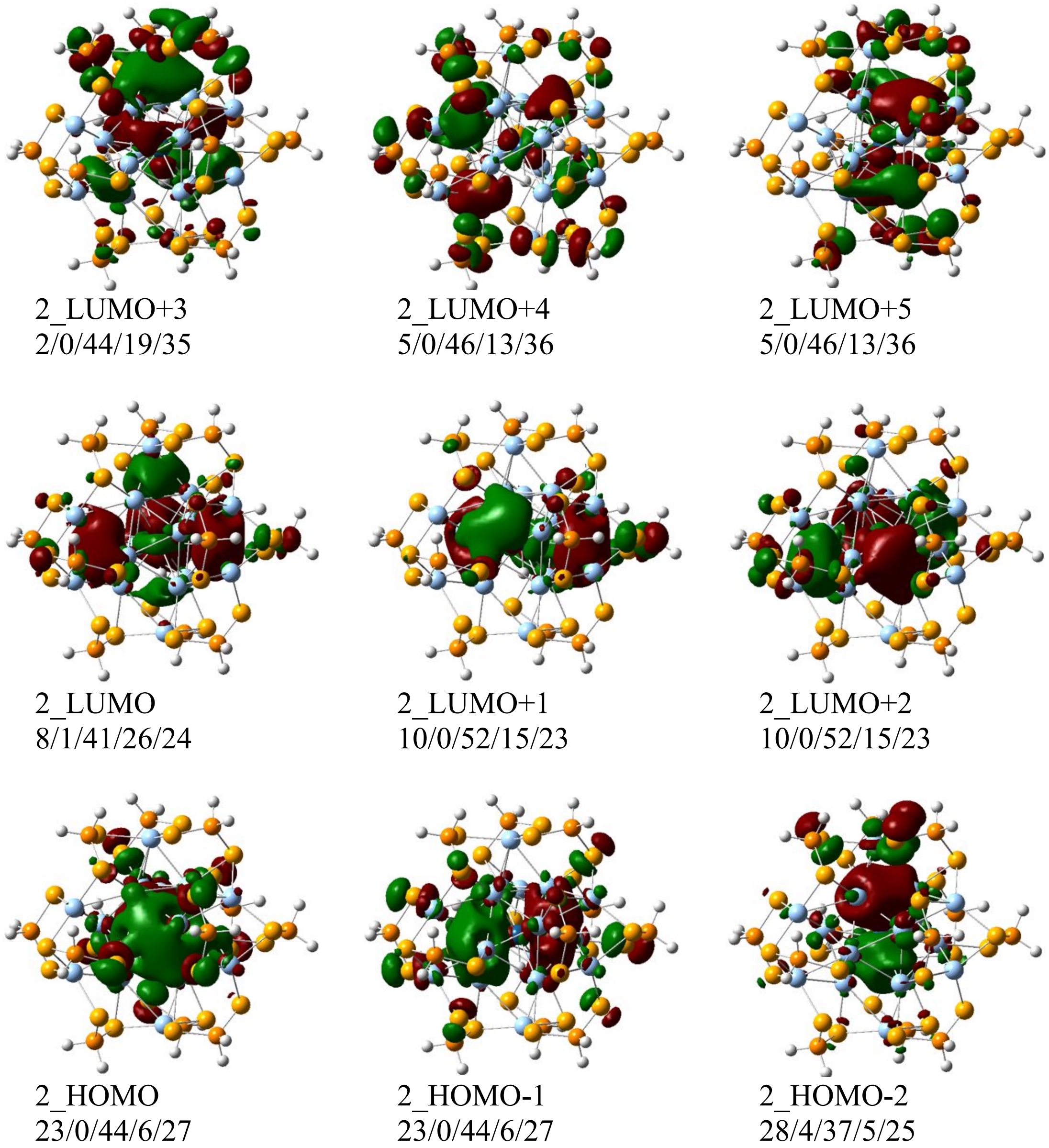

Figure S30. Kohn-Sham frontier orbitals of **2**. Atomic contributions (in %) are given in the order: Ir/H/$Ag_{icosahedron}$/$Ag_{capping}$/ligands.

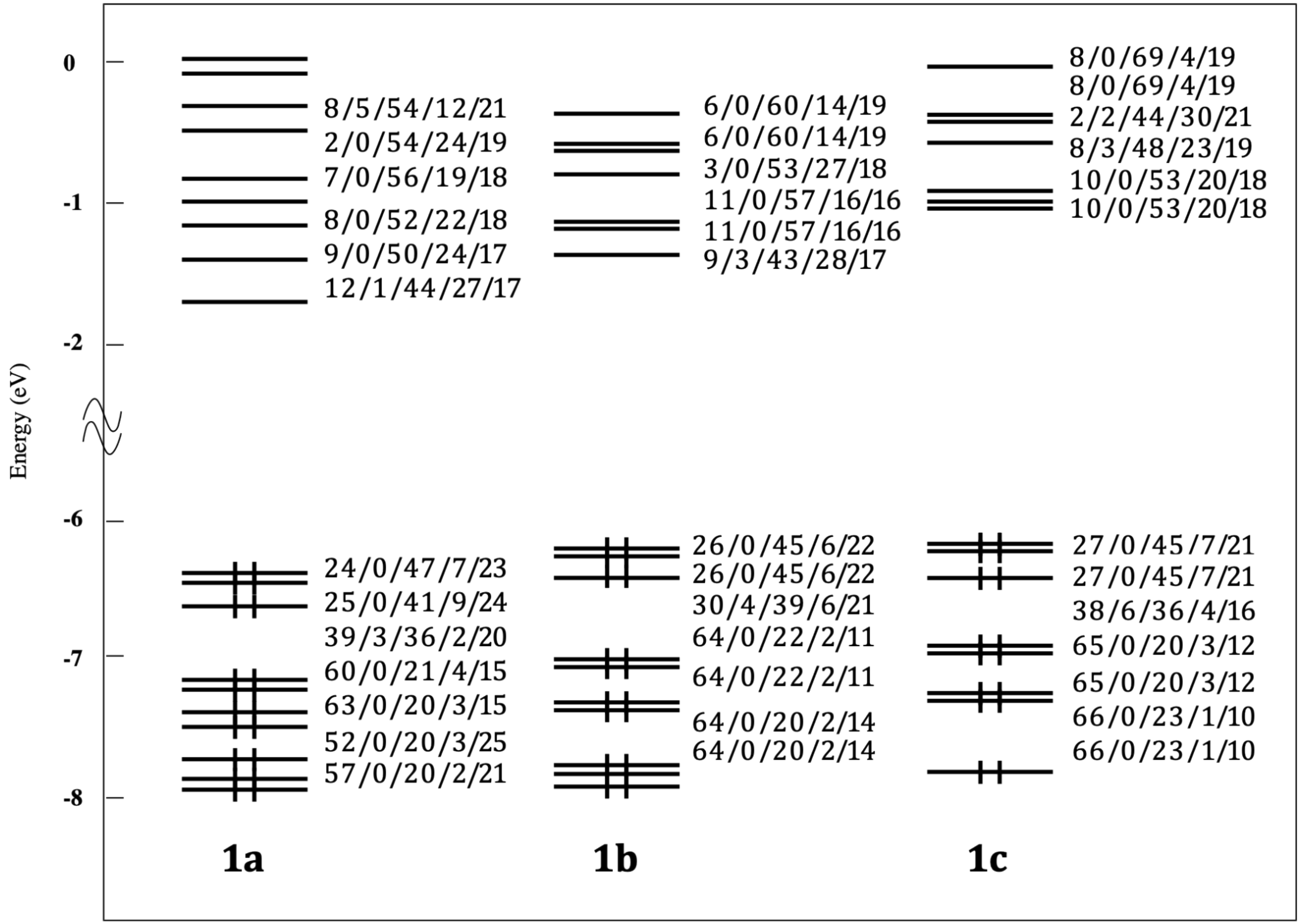

Figure S31. The Kohn-Sham MO diagrams of **1a**-**1c**, with the orbital composition (in %) given in the order: Ir/H/$Ag_{icosahedron}$/$Ag_{capping}$/ligands (CAM-B3LYP/Def2TZVP calculations).

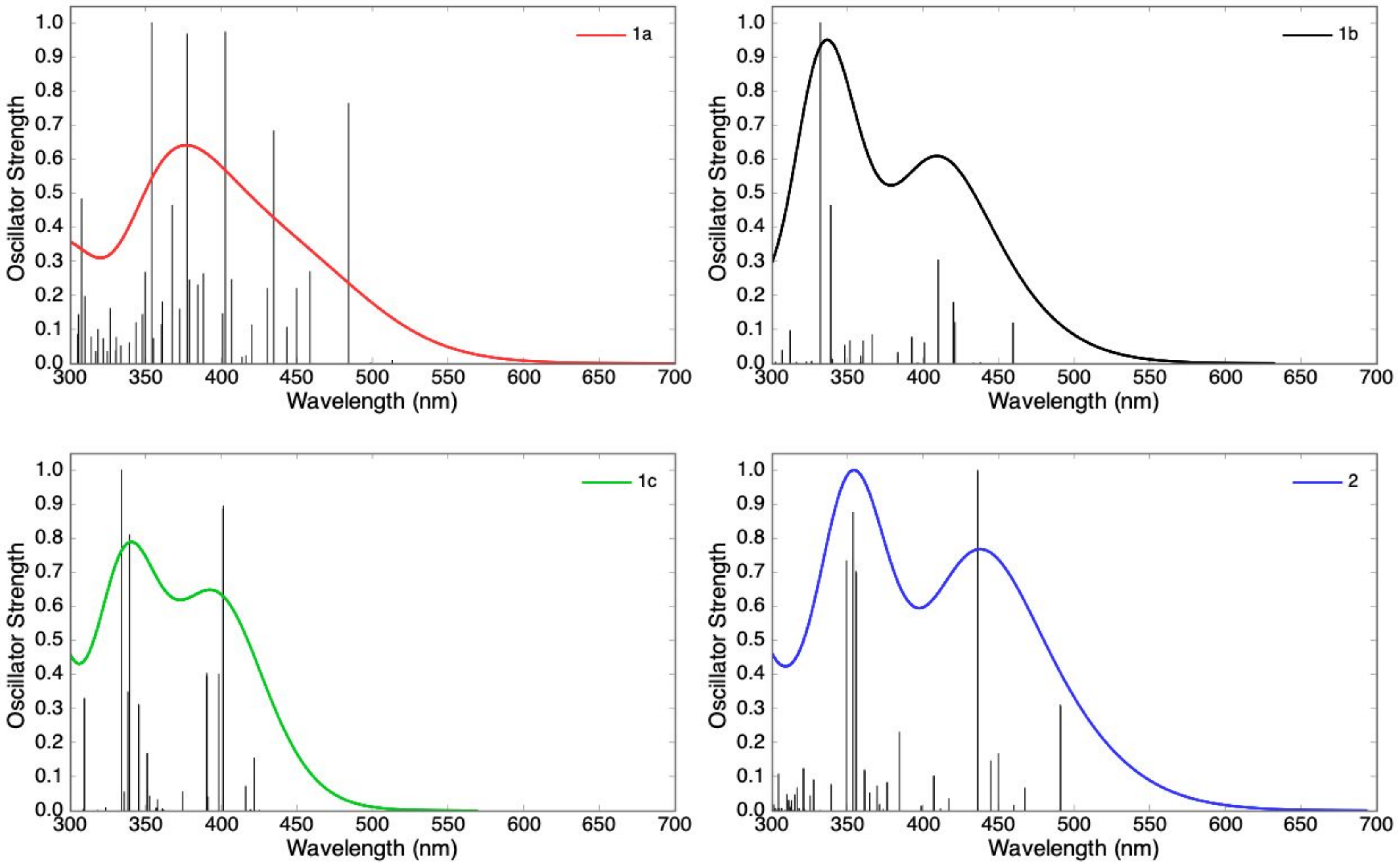

Figure S32. The TD-DFT-computed UV-vis absorption spectra of **1a**, **1b**, **1c** and **2,** with the normalized oscillator strengths from which they have been simulated with Gaussians full width at half maximum of 1000 cm^{-1}.

Table S1. Summary of electronic structure parameters for **1a**, **1b**, **1c** and **2**

	**R1**	**O1**	**O2**	**O3**	**O4**	**O5**	**O6**	**Gap**
**1a**	-2.16	-0.13	0.23	0.42	0.69	1.03	1.27	2.03
**1b**	-2.24	-0.10	0.20	0.42	0.70	1.11	1.26	2.14
**1c**	-1.82	0.28	0.83	1.27				2.10
**2**	-	-0.51	-0.05	0.26	0.61	0.91	1.24	-

Table S2. Selected X-ray crystallographic data of **1a**, **1b**, **1c** and **2**

Compound	**1**	**1b**	**1c**	**2**
CCDC no.	2539362	2539363	2539364	2539365
Chemical formula	$C_{72}H_{169}Ag_{20}IrO_{24}P_{12}S_{24}$	$C_{72}H_{168}Ag_{20}IrO_{24}P_{12}S_{24}$	$C_{146}H_{124}Ag_{20}Cl_4IrP_{12}S_{24}$	$C_{72}H_{169}Ag_{20}IrO_{24}P_{12}Se_{24}$
Formula weight	4909.74	4908.73	5510.92	6035.34
Wavelength, Å	0.71073	0.71073	0.71073	0.71073
Crystal System	Triclinic	Hexagonal	Monoclinic	Triclinic
Space group	$P\bar{1}$	$P6_3$	$P2/c$	$P\bar{1}$
a, Å	15.6233(15)	18.6478(9)	31.451(4)	16.716(3)
b, Å	18.082(2)	18.6478(9)	16.6878(19)	17.500(5)
c, Å	29.254(3)	25.8092(19)	31.411(4)	27.513(5)
α, deg.	82.515(2)	90	90	89.232(6)
β, deg.	77.682(2)	90	91.187(2)	87.845(5)
γ, deg.	67.220(2)	120	90	71.884(4)
V, $Å^3$	7433.7(14)	7772.5(9)	16482(3)	7644(3)
Z	2	2	4	2
Temperature, K	100(2)	296(2)	100(2)	100(2)
ρ_{calcd}, g/cm^3	2.193	2.097	2.221	2.622
μ, mm^{-1}	3.974	3.801	3.651	9.251
θ_{max}, deg.	26.431	24.996	25.000	25.000
Completeness, %	98.7	100	99.9	98.9
Reflection collected / unique	65917 / 29975 [R(int) = 0.0231]	43950 / 9153 [R(int) = 0.0916]	124830 / 29025 [R(int) = 0.0579]	47841 / 26610 [R(int) = 0.0817]
Restraints / parameters	454 / 1444	199 / 483	900 / 1893	774 / 1454
$^aR1$, $^bwR2$ [I > 2σ(I)]	0.0281, 0.0592	0.0631, 0.1395	0.0511, 0.1060	0.0964, 0.2263
$^aR1$, $^bwR2$ (all data)	0.0343, 0.0621	0.1521, 0.2025	0.0676, 0.1140	0.1752, 0.2684
GOF	1.077	1.008	1.118	1.024
Largest diff. peak and hole, e/Å^3	1.774 and -1.584	0.906 and -1.389	2.490 and -2.043	2.199 and -2.667

$^a R1 = \Sigma \left| |F_o| - |Fc| \right| / \Sigma |F_o|$. $^b wR2 = \{\Sigma[w(F_o^2 - F_c^2)^2]/\Sigma[w(F_o^2)^2]\}^{1/2}$.

Table S3. Atomic coordinates of the DFT-optimized structure of **1a, 1b, 1c, and 2**

1a

Ir	0.039656	-0.053349	-0.095129
H	0.428040	-1.049508	-1.382466
Ag	0.732901	0.117880	-2.983651
Ag	-1.563596	-1.785739	-1.686216
Ag	-1.800094	1.187421	-1.873007
Ag	-2.738782	-0.162370	0.610719
Ag	-1.253909	2.337858	0.883497
Ag	0.828055	2.440541	-1.265252
Ag	2.741587	0.197378	-0.726616
Ag	1.342615	-2.672726	-0.577927
Ag	-0.928530	-2.403204	1.262102
Ag	-0.639876	0.124515	2.727937
Ag	1.523206	1.776609	1.558035
Ag	1.816413	-1.148782	1.849728
Ag	-4.433442	-0.484528	-1.837990
Ag	-3.688515	-3.102740	-0.034708
Ag	-3.286360	-1.787543	3.164912
Ag	-1.783074	4.252532	-1.413528
Ag	0.964919	4.736650	0.613637
Ag	3.763126	-2.068559	-2.555278
Ag	4.234176	-2.341432	0.607189
Ag	4.375809	0.751404	1.740169
S	1.799810	-5.169688	-0.473686
S	5.033152	-3.985012	-1.275567
S	-1.975402	-3.482612	-3.551167
S	-5.202537	-2.932863	-2.218178
S	-2.078164	-5.085258	0.499409
S	-1.601486	-3.688475	3.660097
S	-5.457682	-2.725412	1.904216
S	-5.328450	0.532341	0.434588
S	-3.699655	1.162068	-3.725397
S	-4.298930	3.632731	-1.295780
S	1.971746	-3.011678	-4.103702
S	1.734995	0.306530	-5.282013
S	6.354169	-0.709832	0.685891
S	4.795227	0.309440	-2.368298
S	3.355300	-2.984208	3.013328

S	3.909991	0.267865	4.237918
S	-3.807570	0.559482	4.019691
S	-0.587829	0.049512	5.290491
S	2.023778	4.033167	2.872044
S	4.783868	3.181845	0.867672
S	-2.697485	3.864047	2.369873
S	-1.116031	6.228230	0.254930
S	2.349328	4.595062	-1.639645
S	-0.519536	4.481830	-3.668379
P	3.535958	-5.373526	-1.450716
P	-3.962080	-3.438766	-3.768723
P	-2.270324	-5.218815	2.503814
P	-6.318954	-0.958219	1.397672
P	-4.586567	2.914006	-3.140871
P	1.860221	-1.674532	-5.622154
P	6.425024	0.120539	-1.158377
P	4.329758	-1.693416	4.260016
P	-2.448887	0.766447	5.512540
P	4.016129	4.169835	2.429154
P	-2.172615	5.746014	1.942548
P	1.100149	5.463548	-3.005308
H	4.107341	-6.620676	-1.084467
H	3.327308	-5.562981	-2.843032
H	-4.405280	-4.710326	-4.220704
H	-4.346953	-2.622532	-4.866477
H	-3.609561	-5.536792	2.859188
H	-1.596197	-6.393322	2.929830
H	-7.498133	-1.259589	0.666959
H	-6.858393	-0.390246	2.580190
H	-5.982217	2.784537	-3.370593
H	-4.232697	3.855611	-4.143499
H	2.935840	-1.884068	-6.528168
H	0.766422	-2.110510	-6.417258
H	6.980158	1.420317	-1.035319
H	7.436186	-0.549486	-1.895081
H	4.111325	-2.179839	5.575558
H	5.725814	-1.922852	4.119951
H	-2.424250	2.155488	5.812564
H	-2.987049	0.255882	6.726985
H	4.337473	5.546691	2.295978

H	4.690307	3.847451	3.636875
H	-1.466383	6.338727	3.024048
H	-3.334294	6.563686	1.913370
H	0.755213	6.758088	-2.529346
H	1.907873	5.799003	-4.123785

1b

Ir	0.000164	0.000153	-0.102743
H	0.000706	-0.000332	-1.751604
Ag	1.713052	-0.135779	2.218117
Ag	-0.740559	1.552947	2.216709
Ag	-0.976499	-1.416732	2.216311
Ag	2.801001	-0.262365	-0.505343
Ag	-1.172678	2.556702	-0.507877
Ag	-1.627053	-2.293874	-0.508560
Ag	1.580225	2.302704	0.564704
Ag	-2.784452	0.217575	0.562156
Ag	1.203419	-2.519713	0.564679
Ag	1.111906	1.688342	-2.214246
Ag	-2.016331	0.117746	-2.216329
Ag	0.909228	-1.805714	-2.214421
Ag	3.983305	2.309442	-1.623172
Ag	-3.990198	2.294009	-1.628423
Ag	0.009764	-4.602818	-1.626532
Ag	4.263648	1.671311	1.703714
Ag	-3.580787	2.857386	1.698920
Ag	-0.685596	-4.529348	1.700200
Ag	0.002799	0.000475	-4.695348
Ag	-0.003339	-0.000798	4.725994
S	4.016372	-0.150719	3.529603
S	-1.881391	3.554905	3.526459
S	-2.142027	-3.405361	3.524915
S	5.099295	-1.427199	-0.518115
S	-1.313375	5.129349	-0.522241
S	-3.784744	-3.702090	-0.524352
S	3.018719	4.010126	1.981616
S	-4.983876	0.609797	1.976444
S	1.961926	-4.620159	1.979720
S	1.002968	2.398984	-4.734421
S	-2.574350	-0.333981	-4.736911

S	1.580772	-2.064748	-4.734284
S	3.074805	4.729638	-1.471775
S	6.118975	1.937777	-0.103362
S	4.079430	0.928217	-3.841524
S	-5.632105	0.297087	-1.477182
S	-4.737741	4.330025	-0.110119
S	-2.840506	3.066236	-3.846386
S	2.559978	-5.026294	-1.473438
S	-1.380837	-6.268026	-0.108825
S	-1.232148	-3.993947	-3.845823
S	1.674629	1.952752	5.128440
S	-2.534831	0.475670	5.125142
S	0.848708	-2.431259	5.128375
P	3.368148	0.883892	5.173960
P	-2.455567	2.476681	5.170322
P	-0.924136	-3.362665	5.170968
P	6.492389	-0.001259	-0.647355
P	-3.244726	5.622656	-0.653506
P	-3.246461	-5.621561	-0.653587
P	3.690301	5.117251	0.401096
P	-6.277098	0.637758	0.394822
P	2.585889	-5.754484	0.398908
P	3.003911	2.091368	-5.066640
P	-3.308800	1.552254	-5.070811
P	0.314420	-3.643686	-5.068884
H	3.353893	-0.071776	6.224654
H	4.425192	1.734185	5.593790
H	-1.622072	2.942291	6.221978
H	-3.720865	2.967381	5.588449
H	-1.745872	-2.872688	6.220790
H	-0.717564	-4.703595	5.590279
H	7.030260	0.077693	-1.960269
H	7.640524	-0.406343	0.084580
H	-3.581077	6.048302	-1.966879
H	-3.468527	6.819922	0.077591
H	-3.446554	-6.126750	-1.966590
H	-4.171776	-6.413264	0.077885
H	3.366628	6.466999	0.699089
H	5.109645	5.162106	0.467963
H	-7.285095	-0.316528	0.692564

H	-7.024827	1.845085	0.460278
H	3.916126	-6.149985	0.697952
H	1.914355	-7.005814	0.464238
H	3.153748	1.612157	-6.394889
H	3.561455	3.392388	-5.182300
H	-2.968022	1.920960	-6.399068
H	-4.714190	1.384141	-5.187168
H	-0.173810	-3.533280	-6.397715
H	1.162498	-4.777005	-5.183988

1c

Ir	-0.000232	-0.000198	-0.105869
H	-0.004027	0.002791	-1.782351
Ag	-1.613496	-1.188365	-2.208945
Ag	2.635484	-0.978766	0.564652
Ag	0.426666	-2.770517	-0.559234
Ag	1.820747	-0.798386	-2.230177
Ag	-2.161454	-1.791075	0.591814
Ag	2.178947	1.758606	-0.569541
Ag	0.271460	-1.705362	2.193864
Ag	-0.245030	1.984130	-2.217864
Ag	-1.591898	0.626528	2.205250
Ag	-0.463971	2.769036	0.582859
Ag	1.357512	1.074363	2.190220
Ag	-2.617602	1.012289	-0.541342
Ag	2.385227	-3.827603	1.522925
Ag	-0.026694	0.005686	-4.639768
Ag	-2.184346	-4.063896	-1.519032
Ag	0.027237	-0.004112	4.735785
Ag	4.598325	0.152433	-1.552464
Ag	-2.443969	3.918026	-1.507722
Ag	-4.493533	-0.144823	1.563999
Ag	2.137251	3.965472	1.532831
S	-3.115654	-2.774911	-3.623246
S	5.321661	-0.980682	0.655449
S	0.173639	-5.129020	-1.782839
S	3.938866	-1.288378	-3.660036
S	-3.510624	-4.113855	0.698141
S	4.338162	2.727859	-1.800036
S	-0.085002	-2.600763	4.676508

S	-0.882060	4.077636	-3.626890
S	-2.166951	1.389138	4.691409
S	-1.803484	5.096600	0.701506
S	2.332110	1.196108	4.665933
S	-4.546053	2.406763	-1.750387
S	0.384959	-5.483818	1.719336
S	3.307955	-2.745398	3.715613
S	4.062584	-4.086596	-0.463904
S	2.103169	1.458784	-4.935517
S	-2.351244	1.124587	-4.915404
S	0.167485	-2.565017	-4.934267
S	-5.576258	-1.470389	-0.411601
S	4.575477	3.065377	1.702711
S	-3.996726	-1.480154	3.754749
S	1.507650	5.560179	-0.440935
S	0.756859	4.214925	3.738297
S	-4.928384	2.415917	1.755303
P	-1.756372	-3.110059	-5.118594
P	5.572746	-2.968302	0.230024
P	0.431900	-6.288223	-0.114090
P	3.533785	0.065207	-5.143357
P	-5.359603	-3.336268	0.283771
P	5.228903	3.520728	-0.134533
P	1.836687	-3.237467	4.984131
P	-1.863861	3.062778	-5.110852
P	-3.675224	0.042997	5.016821
P	-0.209142	6.307841	0.270298
P	1.927500	3.177033	4.990416
P	-5.664880	2.768384	-0.073266
H	-1.789478	-4.492116	-5.440662
H	-2.342241	-2.544861	-6.282374
H	6.669366	-3.091683	-0.663883
H	6.126891	-3.521524	1.415254
H	1.643350	-6.993277	-0.345177
H	-0.510993	-7.349132	-0.164763
H	3.323868	-0.718071	-6.309307
H	4.744391	0.728191	-5.474688
H	-6.107842	-3.537360	1.474403
H	-6.021249	-4.225323	-0.604285
H	5.230224	4.923871	-0.356608

H	6.618735	3.236178	-0.201997
H	1.733400	-4.645782	5.140231
H	2.226395	-2.849548	6.293538
H	-1.091150	3.283992	-6.281773
H	-3.047195	3.780779	-5.425642
H	-4.842054	0.836266	5.182925
H	-3.520786	-0.485611	6.325872
H	0.003036	7.057529	1.458091
H	-0.655943	7.324752	-0.614642
H	3.200803	3.789045	5.140526
H	1.407547	3.312165	6.305069
H	-6.882213	2.069112	-0.290651
H	-6.114438	4.114584	-0.126175

2

Ir	0.000454	0.000335	-0.075980
Ag	-1.552716	-1.309125	-2.201639
Ag	-0.392729	2.000376	-2.192360
Ag	1.900783	-0.651807	-2.223533
Ag	0.335211	-1.713620	2.220876
Ag	0.584203	-2.750727	-0.518851
Ag	-2.112465	-1.815377	0.583657
Ag	-2.681744	0.872254	-0.473004
Ag	-0.507739	2.736075	0.596409
Ag	2.088631	1.889828	-0.501554
Ag	2.633499	-0.930471	0.553718
Ag	1.331415	1.114720	2.228866
Ag	-1.616021	0.562762	2.248527
Ag	-0.033654	0.020722	-4.702110
Ag	-2.800671	3.696365	-1.570865
Ag	-3.179781	3.261380	1.751511
Ag	-1.815276	-4.248466	-1.610124
Ag	-1.228891	-4.406623	1.701759
Ag	4.581061	0.578072	-1.630629
Ag	4.441775	1.116350	1.687090
Ag	0.034711	-0.023971	4.722730
H	-0.009850	0.004340	-1.727233
Se	-3.519340	2.300239	-3.791335
Se	0.138828	2.715257	-4.774763
Se	2.211882	-1.475642	-4.810510

Se	3.695400	1.897081	-3.838865
Se	-2.454934	-1.173289	-4.776348
Se	-0.262416	-4.146610	-3.841063
Se	-4.390811	-3.445694	-1.595661
Se	-3.741360	-3.364204	2.175762
Se	5.178430	-2.052301	-1.658890
Se	4.801087	-1.586486	2.121648
Se	6.214134	2.141956	-0.068669
Se	3.179159	4.309522	-1.012863
Se	-0.821160	5.528750	-1.582199
Se	-1.016509	4.920690	2.192566
Se	-4.958298	4.310055	0.016952
Se	-5.327623	0.606192	-0.958130
Se	-0.473941	2.624247	5.099584
Se	-3.815518	1.465974	3.688568
Se	0.660999	-4.088400	3.627874
Se	-2.000654	-1.793271	5.093551
Se	3.233892	2.566324	3.642501
Se	2.587922	-0.906140	5.064464
Se	-1.257425	-6.448212	-0.059886
Se	2.126797	-4.906580	-1.047949
P	-2.018711	3.109744	-5.145765
P	3.626906	0.196424	-5.197766
P	-1.720314	-3.234486	-5.177190
P	-4.824712	-4.158399	0.415819
P	6.022588	-2.099004	0.347720
P	5.211590	4.103431	-0.304650
P	-1.194099	6.256709	0.435860
P	-6.163874	2.471196	-0.252539
P	-2.615951	2.354117	5.318506
P	-0.693459	-3.515520	5.277623
P	3.427190	1.084230	5.270560
P	0.937586	-6.565947	-0.335753
H	-2.323638	2.705757	-6.472812
H	-2.135970	4.519255	-5.275817
H	3.411239	0.666780	-6.520458
H	4.904218	-0.406402	-5.347625
H	-2.883253	-4.038727	-5.312105
H	-1.224258	-3.280096	-6.507259
H	-4.786018	-5.578314	0.459091

H	-6.204428	-3.935820	0.664803
H	6.524071	-3.407399	0.575292
H	7.231318	-1.353252	0.395836
H	6.076592	4.876118	-1.124811
H	5.387899	4.768843	0.937648
H	-0.312209	7.343556	0.672915
H	-2.445032	6.928091	0.498691
H	-7.248980	2.848526	-1.088101
H	-6.850616	2.286872	0.976940
H	-2.927727	1.589912	6.473951
H	-3.197073	3.602479	5.664112
H	0.134624	-3.418508	6.427018
H	-1.480598	-4.647772	5.615253
H	4.803720	0.964178	5.596605
H	2.937407	1.736929	6.432599
H	1.445753	-7.065524	0.892806
H	1.155074	-7.694234	-1.170995
